# Transcriptomic and Metabolomic Analyses Reveals That Exogenous Methyl Jasmonate Regulates Galanthamine Biosynthesis in *Lycoris longituba* Seedlings

**DOI:** 10.3389/fpls.2021.713795

**Published:** 2021-09-30

**Authors:** Qingzhu Li, Junxu Xu, Yuhong Zheng, Yongchun Zhang, Youming Cai

**Affiliations:** ^1^Forestry and Pomology Research Institute, Shanghai Academy of Agricultural Sciences, Shanghai, China; ^2^Shanghai Co-Elite Agricultural Sci-Tech (Group) Co., Ltd., Shanghai, China; ^3^Institute of Botany, Jiangsu Province and Chinese Academy of Sciences, Nanjing Botanical Garden, Memorial Sun Yat-sen, Nanjing, China

**Keywords:** *Lycoris longituba*, methyl jasmonate(MeJA), galanthamine biosynthesis, transcriptomics, metabolome, tyrosine decarboxylase

## Abstract

The Amaryllidaceae alkaloid galanthamine (Gal) in *Lycoris longituba* is a secondary metabolite that has been used to treat Alzheimer’s disease. Plant secondary metabolism is affected by methyl jasmonate (MeJA) exposure, although the regulatory mechanisms of MeJA on *L. longituba* seedlings remains largely unknown. In the present study, 75, 150, and 300 μM MeJA were used as treatments on *L. longituba* seedlings for 7, 14, 21, and 28 days, while 0 μM MeJA was used as the control (MJ-0). The effect of exogenous MeJA on Gal synthesis in *L. longituba* was then investigated using transcriptomic sequencing and metabolite profiling via GC-MS and LC-MS analysis. Galanthamine (Gal), lycorine (Lyc), and lycoramine (Lycm) abundances were 2. 71-, 2. 01-, and 2.85-fold higher in 75 μM MeJA (MJ-75) treatment plants compared to MJ-0 treatment plants after 7 days of cultivation. Transcriptomic analysis further showed that MJ-75 treatment significantly induced the expression of norbelladine synthase (NBS) and norbelladine 4′-O-methyltransferase (OMT), which are involved in the Gal biosynthesis pathway. In addition, increased expression was observed in MJ-75 treatment plants for genes in the JA synthesis and JA signaling pathways including those of allene oxide cyclase (AOC), 12-oxo-phytodienoic acid reductase (OPR), jasmonic acid amino acid synthase (JAR), and transcription factor MYC. The *L. longituba* tyrosine decarboxylase (LlTYDC) enzyme was identified and proposed to be involved in the Gal biosynthetic pathway. Metabolomics results demonstrated that the accumulation of Amaryllidaceae alkaloids, and especially alkaloids in the Gal biosynthesis pathway, could be induced by MJ-75 treatment. Interestingly, metabolites in the JA synthesis pathway were also affected by MeJA treatment. Overall, this multi-omics study suggests that both the JA synthesis/JA signaling and Gal biosynthesis pathways were affected by exogenous MeJA treatment. This comprehensive study of gene expression and metabolite contents can help us better understand the molecular mechanisms underlying MeJA-mediated Gal biosynthesis in *L. longituba*.

## Introduction

Plants of the *Lycoris* genus, and especially *Lycoris longituba*, are ornamental flowers and traditional Chinese medicinal herbs used to treat sore throats, carbuncles, and edema (Howes and Houghton, 2003; Wu et al., 2008). The major active ingredients of *L. longituba* are Amaryllidaceae alkaloids (Guo et al., 2014; Li Q. Z. et al., 2018). Six hundred Amaryllidaceae alkaloids, including galanthamine (Gal), lycoramine (Lycm), and lycorine (Lyc), have been identified from the Amaryllidaceae plant family (Jin and Yao, 2019; Desgagné-Penix, 2020). Their pharmacological uses have been reported to include acetylcholinesterase inhibition, cytotoxicity, antibacterial effects, antiviral effects, anti-inflammatory activities, and anticancer effects, among others (Liu et al., 2004; Kornienko and Evidente, 2008; Wang et al., 2010; Kilgore et al., 2016; Hotchandani and Desgagne-Penix, 2017; Pellegrino et al., 2018).

Among the Amaryllidaceae alkaloids, Gal has been commercially used to treat Alzheimer’s disease (Repantis et al., 2010). The biosynthetic pathway for Gal has been recently elucidated (Singh and Desgagné-Penix, 2014; Desgagné-Penix, 2020) and comprises several enzymes including phenylalanine ammonia lyase (PAL), cinnamate-4-hydroxylase (C4H), coumarate 3-hydroxylase (C3H), tyrosine decarboxylase (TYDC), norbelladine synthase (NBS), norbelladine 4′-O-methyltransferase (OMT), and noroxomaritidine synthase (CYP96T1), which have all been functionally characterized in different plant species (Kilgore et al., 2014, 2016; Singh and Desgagné-Penix, 2017; Li W. et al., 2018; Singh et al., 2018; Sun et al., 2018; Li et al., 2019c; Wang et al., 2019; Li Q. et al., 2020). In our previous research, we found that LlOMT may play a role in Gal biosynthesis in *L. longituba*. RNA sequencing of the leaves, roots, and bulbs of *L. longituba* was carried out. Genes involved in the Gal metabolic pathway encoding TYDC, PAL, C4H, C3H, NBS, OMT, and CYP96T1 were detected. LlOMT was identified in the proposed Gal biosynthetic pathway. Overexpression of LlOMT could increase the Gal content (Li Q. et al., 2020). Previous studies showed that Gal was accumulated in all the tissue in *Lycoris* including stems, leaves, flowers, seeds, bulbs, and roots with higher accumulation levels in bulbs, roots, seeds, and flowers (Mu et al., 2010; Li W. et al., 2018; Li et al., 2019b). Also, Gal could be detected both from field and tissue culture derived samples (Mu et al., 2010; Li et al., 2019b; Li Q. Z. et al., 2021). The accumulation of Gal tended to increase with age, reaching a higher value in perennial seedlings in *Lycoris chinensis* (Mu et al., 2010). Generally, the accumulation pattern of Gal varied a lot during different growth stages, for example, Gal was highly accumulated at the dormancy stage in *Lycoris sprengeri* (Li et al., 2019b), and was highly accumulated at flower stage in *Lycoris radiata* (Li W. et al., 2018). Dynamic accumulation pattern of Gal across different growth stages could be observed in *Lycoris* (Mu et al., 2010; Li et al., 2019b). Nevertheless, comprehensive understandings of the pathway and Gal accumulation pattern in *Lycoris* are still lacking.

The limited availability of Gal from plant sources has become one of the main barriers to its use (Ferdausi et al., 2019). Consequently, alternative approaches have been explored by researchers to ensure its sustainable production (Pavlov et al., 2007; Ferdausi et al., 2019). For example, *in vitro* cultures have been used to effectively produce Gal (Ivanov et al., 2013; Schumann et al., 2013; Berkov et al., 2014; Saliba et al., 2015). In addition, various elicitors have been used to increase secondary metabolite levels (Jimenez-Garcia et al., 2013; Ji et al., 2019). Among these, methyl jasmonate (MeJA) is one of the most common elicitors used for *in vitro* cultures (Ivanov et al., 2013; Ram et al., 2013; Perassolo et al., 2016; Ptak et al., 2017; Jiao et al., 2018). Indeed, MeJA elicitation has been used to promote Gal accumulation in *Narcissus confusus*, *Leucojum aestivum*, and *Lycoris chinensis* seedlings (Colque et al., 2004; Mu et al., 2009; Ivanov et al., 2013; Ptak et al., 2017; Wang et al., 2017).

The combination of transcriptomic and metabolomic analyses is a powerful tool for comprehensively studying the gene and metabolite networks that respond to different factors (Liu et al., 2017; Wang et al., 2017; Li W. et al., 2020). For example, combined transcriptomic and metabolomic analyses were used to investigate the ethylene-mediated ripening of tomato plants (Alba et al., 2005), flavonoid biosynthesis in *Fagopyrum tataricum* (Li et al., 2019a), and monoterpene biosynthesis in grape berry skins (Li W. et al., 2020). However, the regulatory mechanisms of MeJA in Gal biosynthesis within *L. longituba* remain unclear. In this study, a combination of GC-MS, LC-MS, and transcriptomic analyses were used to investigate the role of MeJA treatment on metabolic and gene expression changes, and particularly in the Gal and JA synthesis pathways. Further, one of the genes in the Gal pathway was characterized and identified as *LlTYDC*. These results increase our understanding of the mechanisms underlying the influence of exogenous MeJA on Gal biosynthesis in *L. longituba*.

## Materials and Methods

### Plant Materials

**FIGURE 1:**
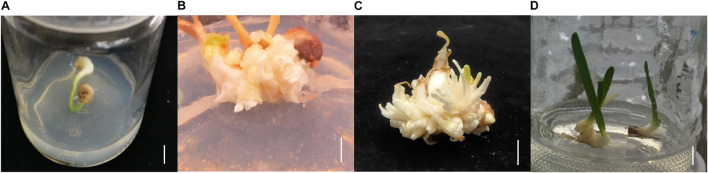
The tissue culture steps of *L. longituba* seedlings. **(A)** Bulblet was formed from seed germination. **(B)** Adventitious bud induction of *L. longituba* bulblet. **(C)** Multiplication of adventitious bud. **(D)** Bulblet expansion and formation of *L. longituba* seedlings. All bars = 1 cm.

*Lycoris longituba* plants were identified by Prof. Zheng Yuhong and grown at the resource nursery at the Shanghai Academy of Agricultural Sciences in China (31.23° N, 121.10° E). *L. longituba* voucher specimens were deposited as we have described previously (Li Q. et al., 2020). Self-pollinated seeds from single plants were collected in September 2018 and seed coats were removed, followed by sterilization in 75% (v/v) ethanol for 2 min and then incubation in 0.5% (m/v) benzyldodecyldimethylammonium bromide (Shanghai Yuanye Bio-Technology Co., Ltd.) for 30 min. The seeds were then washed six times with sterile distilled water and transferred to Murashige and Skoog (MS) solid medium (Beijing Coolaber Technology Co., Ltd.) for seed germination. After 2 months of cultivation and bulblet formation, they were cut into small pieces and transplanted to MS medium supplemented with 30 g/L sucrose, 5.0 mg/L 6-Benzylaminopurine (6-BA), 0.5 mg/L 1-naphthylacetic acid (NAA), 1.0 mg/L forchlorfenuron (CPPU), and 0.5 mg/L thidiazuron (TDZ) (pH = 5.8) to initiate adventitious bud induction. Two months later, adventitious buds were transplanted to MS medium supplemented with 30 g/L of sucrose, 5.0 mg/L 6-BA, and 1.5 mg/L NAA (pH = 5.8) to promote adventitious bud multiplication and growth for another 2 months. Adventitious buds were then transplanted to MS medium supplemented with 40 g/L sucrose, 2.0 mg/L 6-BA, and 0.2 mg/L NAA (pH = 5.8) for bulblet expansion (Figure 1).

Twelve-month-old seedlings exhibiting uniform size were transferred to liquid medium. Then, 1.2 g of bulblets were transferred into 20 mL of MS liquid medium supplemented with 40 g/L sucrose, 2.0 mg/L 6-BA, and 0.2 mg/L NAA, in addition to four different concentrations of MJ (0, 75, 150, and 300 μmol/L). These treatments were identified as MJ-0, MJ-75, MJ-150, and MJ-300, respectively. Liquid culture was maintained in an incubator with shaking at 120 rpm (24 ± 1°C) and incubated for various periods of time (7, 14, 21, and 28 days) under white fluorescent light with a 12 h photoperiod (50 μmol m^–2^ s^–1^). The fresh weights of the control and treated seedlings were then evaluated after 28 days of cultivation. Dry weights were measured by first deactivating enzymes at 105°C and then drying to constant weights at 80°C in an oven. Three independent biological replicates were used for each condition.

### Nitrogen Metabolism-Related Enzyme Activities

Enzyme activities were determined by using enzyme activity measurement kits. Nitrate reductase (YX-W-A800, Sino Best Biological Technology Co., Ltd.), glutamine synthetase (BC0910, Solarbio Life Science), and glutamate synthase (YX-W-A801, Sino Best Biological Technology Co., Ltd.) activities were determined according to individual kit manufacturer’s instructions. Experiments were conducted in three independent biological replicates.

### RNA Extraction, cDNA Library Construction, Sequencing, *de novo* Assembly, and Transcript Annotation

RNA-seq analyses were performed at Shanghai Personal Biotechnology Co., Ltd. (Shanghai, China). Specifically, total RNA was extracted from six samples including three independent biological replicates for the MJ-0 and MJ-75 groups using the TRlzol reagent. Library construction was then conducted as described previously (Li Q. et al., 2020). The six sample libraries were sequenced on the Illumina NovaSeq sequencing platform. Raw data were then quality filtered to remove low-quality reads and clean reads were assembled using the Trinity software program (version 2.5.1) (Grabherr et al., 2011) and aligned to generate unigenes. Annotations were then assigned based on BLAST (version 2.5.0) comparisons to the KEGG (Kanehisa et al., 2012), NCBI NR (Pruitt et al., 2007), eggNOG (Huerta-Cepas et al., 2019), and Swiss-Prot databases (Boutet et al., 2007) using an *E*-value cutoff for identification of 10^–5^ (Mortazavi et al., 2008; Wang et al., 2009). All of the raw data have been deposited in the NCBI Sequence Reads Archive (SRA) under the accession number PRJNA720237.

### Differential Gene Expression Analysis

Unigenes from the MJ-0 and MJ-75 samples were used to calculate reads per kilobase of exon per million mapped reads (RPKM) values. Differentially expressed unigenes were then identified based on *p* ≤ 0.05 and | log2 (fold change)| > 1 thresholds with the DEGseq software package (version 1.32.0) (Wang et al., 2009). The differentially expressed genes (DEGs) were then further investigated based on comparisons to the Gene Ontology (GO; Gene Ontology Consortium, 2015)^1^, KEGG^2^ (Kanehisa et al., 2012), and eggNOG databases^3^ (Huerta-Cepas et al., 2019). Analysis was conducted in three independent biological replicates.

### Reverse Transcription-Quantitative PCR (RT-qPCR) Validation of Gene Expression

RNA samples were reverse-transcribed to generate cDNA and RT-qPCR assays were conducted as described previously (Li Q. et al., 2020). The *actin* gene from *L. longituba* was used as an internal reference for comparison. All of the experiments were conducted with three independent biological replicates. PCR primer information is shown in Supplementary Table 1.

### Alkaloid Extraction and Quantification

Galanthamine (Gal), Lycoramine (Lycm), and Lycorine (Lyc) were purchased from Shanghai TCI Development Co., Ltd., and characterized as described previously (Li Q. Z. et al., 2018). About 0.2 g of plant tissues were freeze-dried and extracted by ultrasonication in 2 ml of 70% HPLC grade ethanol. The mixture was then centrifuged at 12,000 rpm for 10 min to obtain supernatants that were then vacuum-dried and re-dissolved in 1 mL of 0.1% formic acid-acetonitrile (V/V = 95/5) for LC-MS/MS analysis. The analysis and separation conditions were the same as we have described elsewhere (Li Q. et al., 2020). The transition reactions 288→231, 290→189, 288→146 (m/z) were used to quantify Gal, Lycm, and Lyc, respectively. Experiments were conducted with three independent biological replicates.

### Extraction and Quantification of Gal Biosynthetic Pathway Metabolites

L-phenylalanine, trans-cinnamic acid, 4-hydroxycinnamic acid, 4-hydroxybenzaldehyde, 3,4-dihydroxycinnamic acid, 3,4-dihydroxybenzaldehyde, L-tyrosine, tyramine, norbelladine, 4′-O-methylnorbelladine, N-demethylgalanthamine, and galanthamine were extracted and measured using mass spectrometry. About 0.2 g of plant tissues were freeze-dried and extracted by ultrasonication in 2 ml of 70% HPLC grade ethanol. The mixture was then centrifuged at 12,000 rpm for 10 min to obtain the supernatants that were then vacuum-dried and re-dissolved in 1 mL of water-methanol (V:V = 4:1) for LC-MS/MS analysis. Norbelladine and N-demethylgalanthamine were purchased from the Shanghai Puxin Chemical Technology Co., Ltd., and all other chemicals were purchased from Shanghai Yuanye Bio-Technology Co., Ltd. HPLC-grade solvents were obtained from Thermo Fisher Scientific (United States). AB ExionLC (AB Sciex) and a Waters UPLC HSS T3 column (100 mm × 2.1 mm, 1.8 μm) were used to analyze the above metabolites. Separation was conducted using 0.1% formic acid (v/v) (A) and acetonitrile (B) with a 10 min linear gradient of 5–100% B at a flow rate of 0.3 mL/min and elution was monitored with an AB Sciex QTRAP 6500+ mass spectrometer (AB Sciex) operating in positive or negative detection mode. Transition reaction m/z values were used for quantifying L-phenylalanine (166→120), trans-cinnamic acid (147→103), 4-hydroxycinnamic acid (163→119), 4-hydroxybenzaldehyde (123→95), 3,4-dihydroxycinnamic acid (179→135), 3,4-dihydroxybenzaldehyde (137→108), L-tyrosine (182→136), tyramine (138→121), norbelladine (260→138), 4′-O-methylnorbelladine (274→137), and N-demethylgalanthamine (274→213). Experiments were conducted with three independent biological replicates.

### Cloning and Sequence Analysis of the *LlTYDC* Gene

A partial *LlTYDC* gene sequence was obtained from our previous RNA-seq dataset (PRJNA590043). To obtain its full-length sequence, the SMARTer^TM^ RACE cDNA Amplification Kit (Clontech Laboratories, Inc., CA, United States) was used to perform 3′-rapid-amplification of cDNA ends (RACE) according to the manufacturer’s protocols. The coding sequence was then verified by using the primers *LlTYDC*-F3 and *LlTYDC*-R3. PCRs were conducted as follows: 94°C for 5 min (one cycle); 94°C for 30 s, 50°C for 45 s, and 72°C for 93 s (30 cycles); and a final extension for 10 min at 72°C. The PCR products of expected size were purified and ligated into the pMD19-T vector (TaKaRa) for subsequent sequencing. The primers used in these analyses are shown in Supplementary Table 2. The amino acid sequences of TYDCs from different plant species were then aligned using Clustal Omega^4^. Phylogenetic analysis of the 16 TYDC proteins was conducted in MEGA X 10.1^5^ using the neighbor-joining phylogenetic method, and node support was determined by 1,000 bootstrap replicates.

### Localization Analysis of LlTYDC

The *LlTYDC* coding region was cloned in-frame with the green fluorescent protein (GFP) gene in the pBWA(V)HS-35S-NOS vector, and the previously published fluorescent protein marker PIN5-mKate was used as the endoplasmic reticulum (ER) marker (Mravec et al., 2009). *Agrobacterium tumefaciens* (strain GV3101)-mediated infiltration was conducted with 6-week-old *Nicotiana benthamiana* leaves, as described previously (Liu et al., 2016). The fluorescent signals in the inoculated leaves were then examined with confocal microscopy (Nikon C2-ER, Nikon) after 3 days. Experiments were conducted in three independent biological replicates.

### Enzyme Activity Assay of LlTYDC

To express recombinant protein, the *LlTYDC* CD with a GST tag was cloned in-frame in the pGEX-4T-1 vector (GE Healthcare) and transformed into *Escherichia coli* BL21 (DE3) cells. The GST-tag fusion protein was then purified using affinity chromatography with the GST-Trap FF Column following manufacturer’s instructions (GE Healthcare). LlTYDC enzyme activity assays were conducted according to previously described methods (Wang et al., 2019). Briefly, the 150 μL (final volume) solution contained 16 μg of recombinant protein, pyridoxal-5-phosphate (10 mM), and L-tyrosine (20 mM) in Tris–HCl (50 mM, pH 8.0). A GST empty vector was used as the control. Reactions were then incubated at 37°C for 40 min and 300 μL methanol was added to the mixtures to terminate reactions. The extracts were vacuum-dried, re-dissolved in 100 μL of 50% methanol, and the products were measured on a Nexera UHPLC LC-30A system (Shimadzu) with a Waters ACQUITY UPLC HSS T3 (100 mm × 2.1 mm, 1.8 μm) column. Separation was carried out using 0.1% formic acid (v/v) (A) and acetonitrile (B) with a 10 min linear gradient of 5–100% B at a flow rate of 0.4 mL/min. The LC elution was monitored using an API-4000-QTRAP mass spectrometer (AB SCIEX) operating in positive detection mode for tyrosine and tyramine. Identification was based on retention times and MS/MS spectra compared to authentic standards (Shanghai Yuanye Bio-Technology Co., Ltd.). The transition reactions 182→136 and 138→121 (m/z) were used for quantifying tyrosine and tyramine. Experiments were conducted in three independent technical replicates.

### Metabolite Extraction for GC-MS Analysis

To determine metabolite compositions with GC-MS, 20 mg samples were accurately weighed, and 360 μL of cold methanol was added to each sample in addition to 40 μL of 2-chloro-l-phenylalanine (0.3 mg/mL, internal standard). Samples were maintained at −80°C for 2 min and then evenly ground. The mixtures were then ultrasonicated in an ice-water bath for 30 min and 200 μL of chloroform was added to the samples and mixed well. Subsequently, 400 μL of water was added to the mixture. Samples were mixed well and ultrasonicated in an ice-water bath for 30 min followed by centrifugation at 12,000 rpm for 10 min at 4°C. A quality control (QC) sample was prepared by mixing all of the samples to form a pooled sample and was injected into every sixth study sample throughout the experiment. Supernatants were vacuum-dried and 80 μL of methoxylamine hydrochloride (15 mg/mL) in pyridine was added to them, followed by vigorous mixing. The mixture was then incubated at 37°C for 90 min and 80 μL of BSTFA (with 1% TMCS) and 20 μL of n-hexane were added to the mixture, followed by vigorous mixing and derivatization at 70°C for 60 min. The samples were then placed at room temperature for 30 min before GC-MS analysis. Water, methanol, chloroform, pyridine, n-hexane, methoxylamine hydrochloride (97%), and BSTFA with 1% TMCS were purchased from CNW Technologies GmbH (Düsseldorf, Germany). In addition, L-2-chlorophenylalanine was purchased from Shanghai Hengchuang Bio-technology Co., Ltd. (Shanghai, China). Experiments were conducted in six independent biological replicates.

### Metabolite Extraction for LC-MS Analysis

To evaluate metabolites with LC-MS, 80 mg samples were weighed and 20 μL of 2-chloro-l-phenylalanine (0.3 mg/mL, internal standard) along with a 1 mL mixture of methanol and water (7:3, V:V) were added to each sample. Samples were then maintained at −80°C for 2 min and evenly ground. The mixtures were then ultrasonicated in an ice-water bath for 30 min and held at −20°C overnight followed by centrifugation at 13,000 rpm for 10 min at 4°C. A QC sample was prepared by mixing all samples to form a pooled sample that was injected into every sixth study sample throughout the experiment. Then, 150 μL of supernatant was filtered with a 0.22 μm filter and transferred to an LC injection vial and stored at −80°C until LC-MS analysis. Experiments were conducted in six independent biological replicates.

### Instrument Parameters for GC-MS and LC-MS Analyses

The derivatized samples for GC-MS analyses were separated and analyzed using the same instrument conditions as described previously (Ning et al., 2019). The instrument parameters were also set as described previously (Jiao et al., 2018). Mass data were acquired in full-scan mode (50–500 m/z) and the solvent delay time was set to 5 min.

Metabolic profiles from LC-MS analysis were generated using an ACQUITY UHPLC system (Waters Corporation, Milford, MA, United States) with an ACQUITY UPLC HSS T3 (100 mm × 2.1 mm, 1.8 μm) column and an AB SCIEX Triple TOF 6600 plus System (AB SCIEX, Framingham, MA, United States) in both ESI positive and negative ion modes. The elution system comprised water (0.1% formic acid, v/v, A) and acetonitrile (0.1% formic acid, v/v, B). Separation was conducted using 5% B for 0.01 min; 5% B for 2 min; 30% B for 4 min; 50% B for 8 min; 80% B for 10 min; 100% B for 14 min; 100% B for 15 min; 5% B for 15.1 min; and 5% B for 16 min. The flow rate was set at 0.35 mL/min and the column temperature was 45°C. Samples were maintained at 4°C during the analysis and the injection volume was 2 μL. Data acquisition was performed in full scan mode (with m/z ranges from 100 to 1,000) combined with the IDA mode. Mass spectrometry parameters were used as described previously (Liu and Zhang, 2021). An m/z range of 40–1,000 was used for IDA analysis and a collision energy of 35 eV was used.

### Data Analyses

The AnalysisBaseFileConverter 4.0.0 and MS-DIAL software 4.24 (Tsugawa et al., 2015) programs were used to convert the raw GC-MS data and process the data, respectively. The LUG database (Untarget database of GC-MS from Lumingbio) was used to annotate metabolites. The data were then aligned to statistically compare components and all internal standards and any known pseudo-positive peaks were removed. After the RSD of the interior label >0.3 were deleted, all peak areas were normalized by the multi-interior label according to retention time partition periods. Data were log10 transformed and then imported into the “ropls” package for R software 3.6.2. To visualize the metabolic differences among groups, principle components analysis (PCA), and (orthogonal) partial least-squares-discriminant analysis (O)PLS-DA were performed. Ellipses in the score plots of models represent the Hotelling’s T2 regions that are defined as the 95% confidence interval of modeled variation. The data scaling modes of PCA and OPLS-DA analyses were unit variance scaling and Pareto scaling, respectively. The importance of variables in projection (VIP) method ranks the total contribution of each variable to the OPLS-DA model. Variables with VIP > 1 were considered to be relevant for group discrimination. Differential metabolites were then identified according to statistically significant thresholds of VIP values from the OPLS-DA model and *p* values from a two-tailed Student’s *t* test among groups. Metabolites with VIP > 1.0 and *p* < 0.05 were thus considered differential metabolites. The identified metabolites were mapped to pathways according to their annotation in the KEGG database, pathway analysis was performed using MetaboAnalyst 5.0^6^. Pathway enrichment analysis was carried out by calculating the significance *p* value of each pathway through the hypergeometric distribution test method.

The Progenesis QI software program 2.3 (Waters Corporation, Milford, MA, United States) was used to analyze raw LC-MS data. Internal standards were used for data QC. The obtained matrix was further reduced by removing the peak of any missing value (ion intensity = 0) in more than 50% of samples. Metabolites were identified using the Progenesis QI (Waters Corporation, Milford, MA, United States) data processing software program by using public databases including HMDB^7^ (Wishart et al., 2018), LIPID MAPS^8^ (O’Donnell et al., 2019), and self-built databases (Lumingbio). PCA and (O)PLS-DA were conducted to visualize the metabolic profile variation among experimental groups. Differential metabolites were selected based on the same criteria described above for GC-MS and pathway enrichment analysis was carried out by the same method as described above. Metabolite-metabolite correlations between identified metabolites were analyzed by using the Pearson correlation method in R software. The metabolic network was then constructed using the Cytoscape software program 2.8.3 (Cytoscape Consortium, CA, United States). Analysis was conducted in six independent biological replicates.

## Results

### Effects of MJ on the Growth and Alkaloid Accumulations in *L. longituba* Seedlings

**FIGURE 2:**
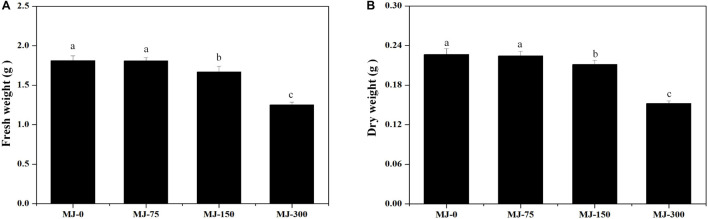
The biomass of *L. longituba* seedlings grown under different MeJA treatments. **(A)** The fresh weight of *L. longituba* seedlings grown under different MeJA treatments. **(B)** The dry weight of *L. longituba* seedlings grown under different MeJA treatments. Error values indicate the standard deviation (SD). Least-significant difference test (LSD, *p* < 0.05) was used to compare the me9.ans. Different letters represent significant differences between groups (*N* = 3, *p* < 0.05).

Clear differences in growth were observed in seedlings grown for 28 days under different MJ treatments. The biomass of seedlings grown with the MJ-75 treatment did not exhibit significant differences with those in the MJ-0 treatment, while the biomass of seedlings grown with the MJ-150 and MJ-300 treatments were lower than in the MJ-0 treatment (Figures 2A,B). The fresh and dry weights also exhibited similar trends (Figures 2A,B).

**FIGURE 3:**
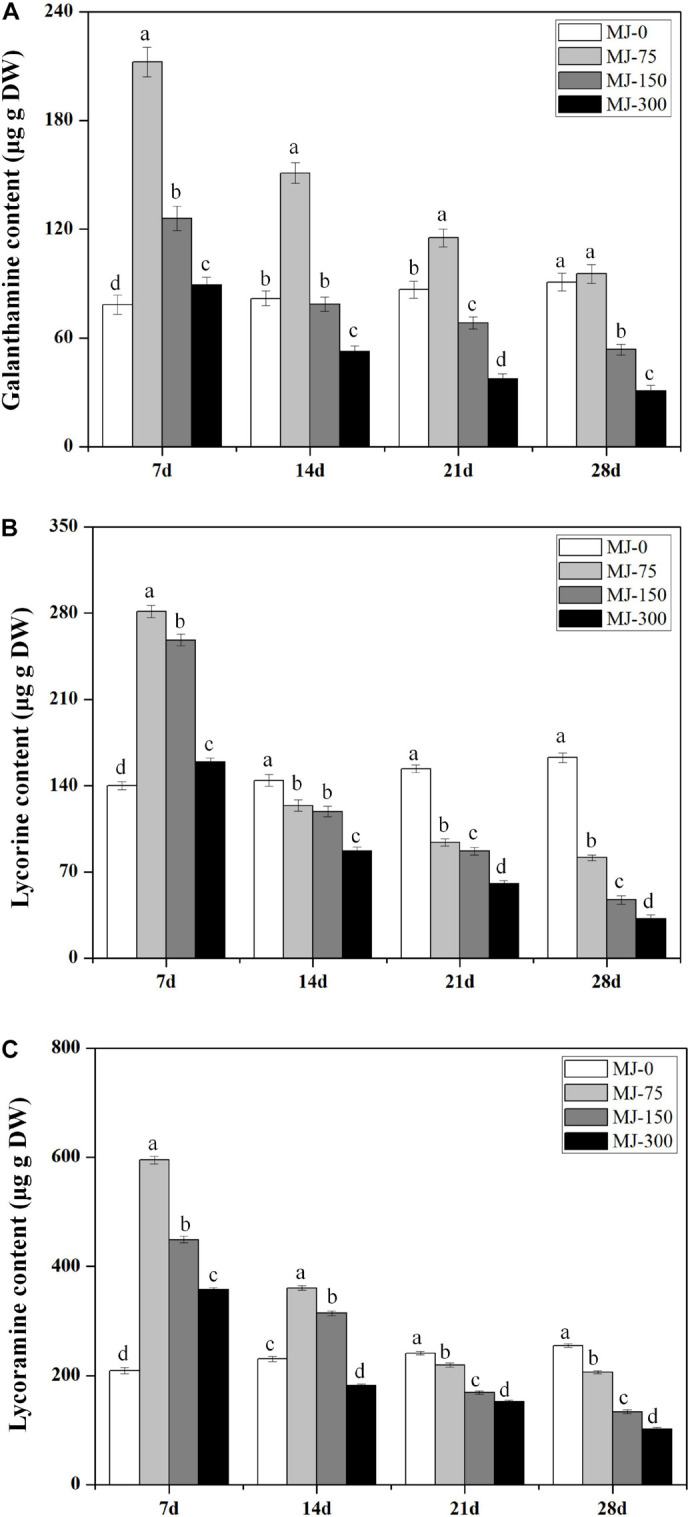
The time course alkaloids accumulations in the seedlings of *L. longituba* treated with different concentrations of MeJA. **(A)** The time course Gal accumulations in the seedlings of *L. longituba* treated with different concentrations of MeJA. **(B)** The time course Lyc accumulations in the seedlings of *L. longituba* treated with different concentrations of MeJA. **(C)** The time course Lycm accumulations in the seedlings of *L. longituba* treated with different concentrations of MeJA. Error values indicate the standard deviation (SD). Least-significant difference test (LSD, *p* < 0.05) was used to compare the means. Different letters represent significant differences between groups (*N* = 3, *p* < 0.05).

Different MJ treatments significantly affected alkaloid (Gal, Lyc, and Lycm) accumulation in the *L. longituba* seedlings across the four MJ treatments at four different time points (Figure 3). Notably, lower concentration treatment of MJ enhanced the accumulation of Gal, Lyc, and Lycm. In addition, the accumulation of Gal, Lyc, and Lycm in the MJ-75 treatment plants were 2. 71-, 2. 01-, and 2.85-fold higher than for MJ-0 after 7 days of culture, respectively (Figures 3A–C). The accumulation of Gal, Lyc, and Lycm in the MJ-150 treatment was 1. 61-, 1. 85-, and 2.15-fold higher than MJ-0 after 7 days of culture, respectively (Figures 3A–C). The accumulation effects of Gal, Lyc, and Lycm could last until days 21, 7, and 14 of the MJ-75 treatments, respectively (Figures 3A–C). The above data indicate that seedling growth and alkaloid accumulations are all improved by MJ-75 treatment after 7 days. Consequently, the samples from this time point were chosen for further experiments.

### Effects of MJ on Nitrogen Metabolism of *L. longituba* Seedlings

**FIGURE 4:**
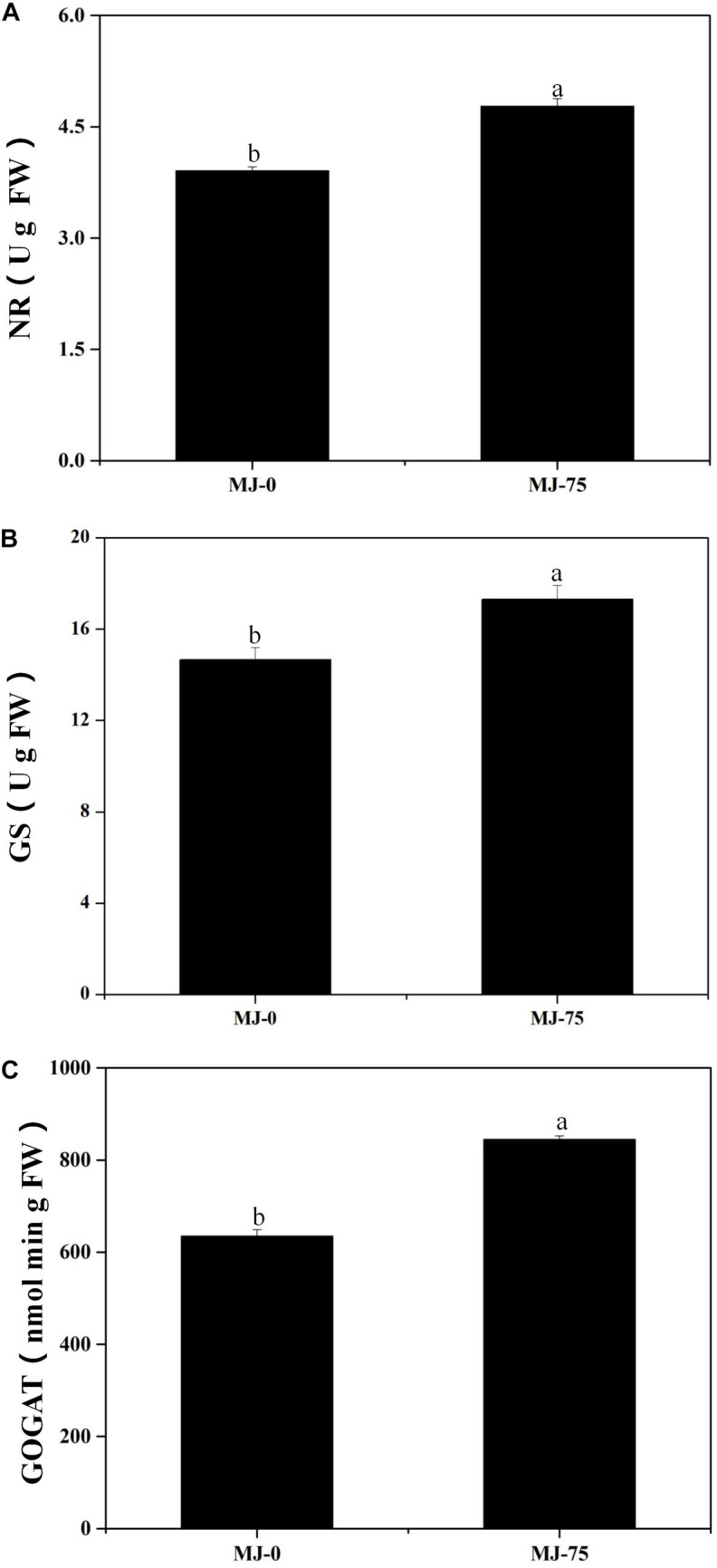
Effects of MeJA on nitrogen metabolism of *L. longituba* seedlings. **(A)** The nitrate reductase (NR) activities in MJ-0 and MJ-75 treatments. **(B)** The glutamine synthetase (GS) activities in MJ-0 and MJ-75 treatments. **(C)** The glutamate synthase (GOGAT) activities in MJ-0 and MJ-75 treatments. Error values indicate the standard deviation (SD). Least-significant difference test (LSD, *p* < 0.05) was used to compare the means. Different letters represent significant differences between groups (*N* = 3, *p* < 0.05).

Nitrate reductase (NR), glutamine synthetase (GS), and glutamate synthase (GOGAT) are the key enzymes involved in *L. longituba* nitrogen metabolism. The NR, GS, and GOGAT activities of the MJ-75 treatment group plants were significantly higher than those for the MJ-0 group at day 7 after MJ treatment (*p* < 0.05). Specifically, the NR, GS, and GOGAT activities of MJ-75 treatment plants were 21.99, 17.99, and 33.15% higher than the MJ-0 plants (Figures 4A–C). Thus, MJ-75 treatment could increase key nitrogen metabolism enzyme activities in *L. longituba* seedlings.

### Illumina Sequencing and Read Assembly

Six total mRNA libraries were generated using Illumina sequencing to establish a transcriptomic database, including three from MJ-0 *L. longituba* seedlings (7 days), and three from MJ-75 *L. longituba* seedlings (7 days). The clean reads were assembled resulting in 610,548 contigs, 416,829 transcripts, and 185,442 unigenes. The mean transcript size was 756 bp, with an N50 of 1,186 bp. The mean unigene size was 614 bp, with an N50 of 873 bp (Supplementary Table 2). The length distributions of the transcripts or unigenes and a summary of the assembly results are shown in Supplementary Figure 1 and Supplementary Table 2, respectively.

### Functional Annotation

BLAST comparisons of the all-unigene datasets against different databases indicated that 54,932 (29.62%) of the unigenes possessed homologous sequences in databases. A total of 52,775 (28.46%), 19,923 (10.74%), 50,289 (27.12%), and 35,831 (19.32%) unigenes had homologs in the nr, KEGG, eggNOG, and Swiss-Prot databases, respectively (Supplementary Tables 3, 18, 19). The distribution of homology to genes in other species was obtained based on nr annotations. A total of 640 plant species exhibited homologous mRNA sequences to those of *L. longituba*, while the annotated unigenes had particularly high similarities to those of *Elaeis guineensis* (19.45%), *Phoenix dactylifera* (19.04%), and *Musa acuminata* subsp. *malaccensis* (6.95%) (Supplementary Figure 2).

**FIGURE 5:**
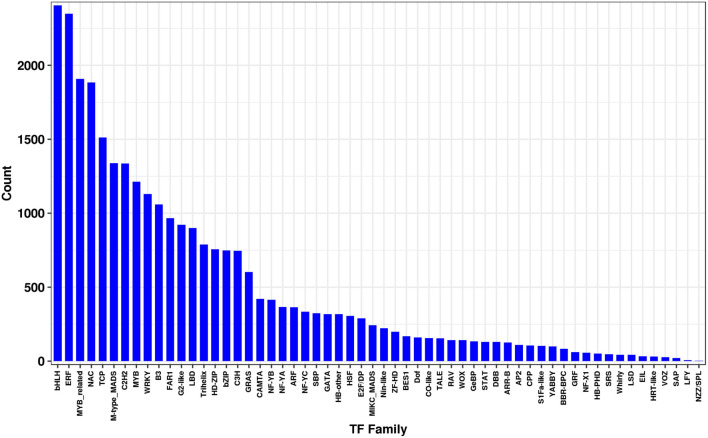
Transcription factors (TF) analysis from the *L. longituba* transcriptome data (*N* = 3).

Transcription factors (TFs), including the MYB, MYB-related, WRKY, and bHLH families, have been reported to play key regulatory roles in plant growth, development, and secondary metabolite biosynthesis (Park et al., 2008; Hichri et al., 2010; Xie et al., 2016; Yin et al., 2017; Yu et al., 2018). To further understand the transcriptional regulation of MeJA-treated *L. longituba*, we used unigene sequences in BLAST searches of the public database PlantTFDB (Guo et al., 2008), and found that a total of 29,056 unigenes encoded putative TFs (15.67%, 58 TF families) (Figure 5). Among these, the bHLH TF family was most abundant (2,406, 8.28%) followed by the ERF (2,348, 8.08%), MYB-related (1,909, 6.57%), NAC (1,885, 6.49%), and TCP (1,512, 5.20%) families (Figure 5 and Supplementary Table 4).

Gene ontology, eggNOG, and KEGG pathway analyses were used to further evaluate the primary biological functions represented within the transcriptomes. Biological processes (BP), cellular components (CC), and molecular functions (MF) categories were used to identify gene product annotations based on GO mapping (Berardini et al., 2004; Supplementary Figure 3). A total of 81,432 unigenes were identified that comprised 50 major functional groups, with binding (GO:0005488), metabolic process (GO:0008152), and cells (GO:0005623) being the most highly represented GO terms in the MF, BP, and CC categories, respectively (Supplementary Figure 3 and Supplementary Table 5). eggNOG mapping revealed that 64,013 annotated unigenes were grouped into 25 eggNOG classification groups. Groups L (replication, recombination, and repair), T (signal transduction mechanisms), and O (post-translational modification, protein turnover, and chaperones) were the three most abundant groups [excluding the R (general function prediction only) and S (function unknown) groups] (Supplementary Table 6 and Supplementary Figure 4). KEGG mapping revealed a total of 23,331 unigenes mapped to 33 major pathways (Supplementary Table 7). The pathways with the greatest unigene representations were signal transduction (1,835 unigenes), carbohydrate metabolism (1,797 unigenes), and folding, sorting, and degradation (1,767 unigenes).

### Differential Expression Analysis

A total of 4,548 differentially expressed genes (DEGs) were detected in the MJ-75/MJ-0 comparison based on thresholds of *p* ≤ 0.05 and a | log2 (fold change)| > 1. Among these, 2,587 unigenes were upregulated and 1,961 were downregulated (Supplementary Figures 5, 6). A total of 512 DEGs could be classified into 79 KEGG secondary metabolic pathways (Supplementary Table 8). Among these, 28 DEG unigenes were involved in the flavonoid biosynthesis pathway, including biosynthetic pathways for phenylpropanoids (23 unigenes) and flavonoids (five unigenes). In addition, 26 DEG unigenes were involved in the cysteine and methionine metabolism pathway. Further, 20 DEG unigenes were involved in the starch and sucrose metabolism pathway, while six were associated with isoquinoline alkaloid biosynthesis. A total of 1,251 differentially expressed TFs comprised 50 TF families and 496 upregulated along with 755 downregulated TFs. The three most abundant TF families were the EFR, NAC, and bHLH families (Supplementary Table 9).

### Gene Expression Analysis of DEGs Involved in *L. longituba* Seedling Gal Biosynthesis

**FIGURE 6:**
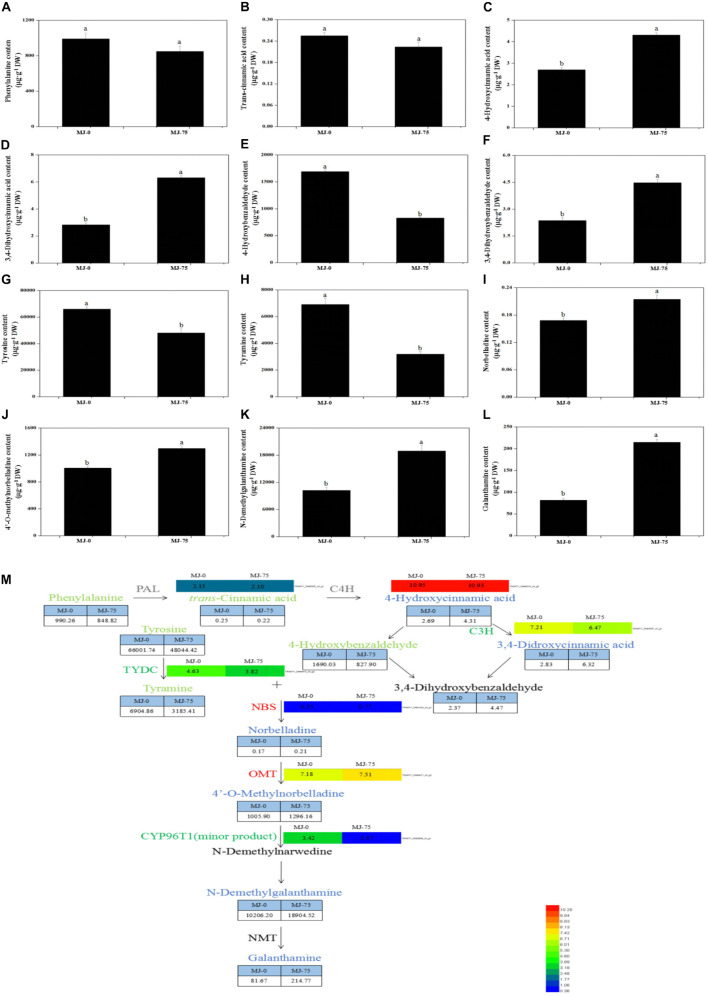
Proposed Gal biosynthesis pathway analysis in the different MJ treated *L. longituba* seedlings. Phenylalanine **(A)**, trans-cinnamic acid **(B)**, 4-hydroxycinnamic acid **(C)**, 3,4-dihydroxycinnamic acid **(D)**, 4-hydroxybenzaldehyde **(E)**, 3,4-dihydroxybenzaldehyde **(F)**, tyrosine **(G)**, tyramine **(H)**, norbelladine **(I)**, 4′-O-methylnorbelladine **(J)**, N-demethylgalanthamine **(K)**, and galanthamine **(L)** contents as measured by LC-MS/MS. Error values indicate the standard deviation (SD). Least-significant difference test (LSD, *p* < 0.05) was used to compare the means. Different letters represent significant differences between groups (*N* = 3, *p* < 0.05). **(M)** Proposed Gal biosynthesis pathway in *L. longituba*. Red or blue colors represent up-regulated genes or metabolites, green colors indicate down-regulated genes or metabolites, black colors represent non-detected metabolites or genes, and gray colors are genes or metabolites that did not change significantly under MJ-75 treatment. Heat maps showed the normalized gene expression values which represent the means ± SD of three biological replicates. Metabolites contents showed the means ± SD by LC-MS/MS. Gene expression values are presented as FPKM normalized log2-transformed counts (*N* = 3, *p* < 0.05).

**FIGURE 7:**
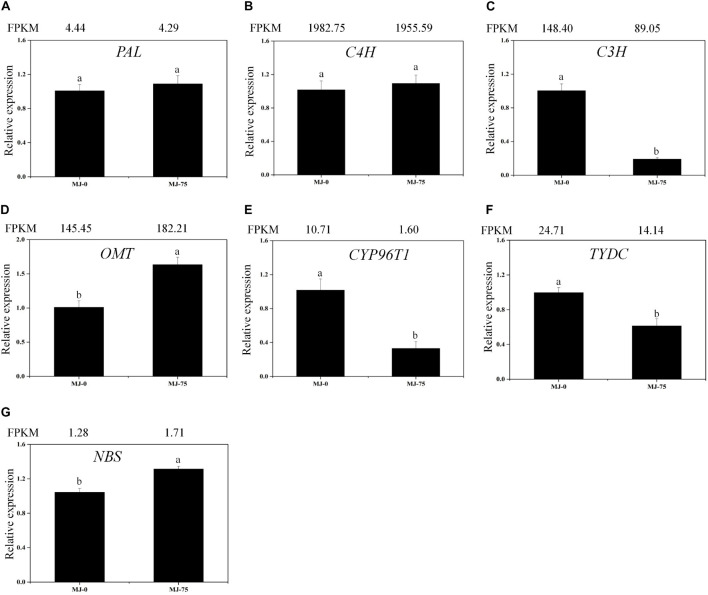
Expression analysis of the DEGs involved in Gal biosynthesis in the different MJ treated *L. longituba* seedlings. RT-qPCR analysis of *PAL* **(A)**, *C4H* **(B)**, *C3H* **(C)**, *OMT* **(D)**, *CYP96T1* **(E)**, *TYDC* **(F)**, and *NBS* **(G)**. The bar chart shows the relative expression levels as measured by RT-qPCR values, while the top vertical axis shows the relative RPKM values. Error values indicate the standard deviation (SD). C3H, *p*-coumarate 3-hydroxylase; C4H, Cinnamate-4-hydroxylase; CYP96T1, noroxomaritidine synthase; DEGs, differentially expressed genes; NBS, norbelladine synthase; OMT, norbelladine 4′-O-methyltransferase; PAL, phenylalanine ammonia; RPKM, Reads Per Kilobase per Million mapped reads; RT-qPCR, real-time quantitative polymerase chain reaction; TYDC, tyrosine decarboxylase. Least-significant difference test (LSD, *p* < 0.05) was used to compare the means. Different letters represent significant differences between groups (*N* = 3, *p* < 0.05).

Local BLAST analyses were conducted to identify putative genes involved in Gal biosynthesis (Supplementary Table 10 and Figure 6). The gene expression results were then confirmed by FPKM digital expression comparisons and through RT-qPCR analysis as we have described previously (Li Q. et al., 2020). The expression profiles of the seven proposed Gal biosynthetic genes were analyzed in MJ-0 and MJ-75 seedlings (Figures 7A–G). *PAL* and *C4H* did not exhibit significant differences between MJ-75 and MJ-0 plants (Figures 7A,B). *C3H, CYP96T1*, and *TYDC* were relatively highly expressed in MJ-0 plants (Figures 7C,E,F), while *OMT* and *NBS* exhibited higher expression in MJ-75 plants compared to MJ-0 plants (Figures 7D,G). The RT-qPCR results matched the FPKM results well.

### Effects of MeJA on Metabolites Involved in *L. longituba* Seedlings Gal Biosynthesis

The 12 metabolites that were suspected to be involved in Gal biosynthetic pathways were analyzed in MJ-0 and MJ-75 samples (Figures 6A–M). Phenylalanine and trans-cinnamic acid did not exhibit significant differences between MJ-75 and MJ-0 treatment plants (Figures 6A,B). In contrast, 4-hydroxycinnamic acid, 3,4-dihydroxycinnamic acid, 3,4-dihydroxybenzaldehyde, norbelladine, 4′-O-methylnorbelladine, N-demethylgalanthamine, and galanthamine were relatively highly prevalent in MJ-75 plants (Figures 6C,D,F,I–L), with 1. 60-, 2. 23-, 1. 89-, 1. 27-, 1. 28-, 1. 85-, 2.63-fold higher values than in MJ-0 treatment plants. In addition, 4-hydroxybenzaldehyde, tyrosine, and tyramine were relatively highly prevalent in MJ-0 treatment plants (Figures 6E,G,H).

### Cloning and Sequence Analysis of *LlTYDC*

The open reading frame (ORF) of the *LlTYDC* gene is 1,551 bp in length and encodes a protein of 517 amino acids. The molecular weight of LlTYDC is approximately 57.50 kDa and exhibits an isoelectric point (pI) of 6.39 (Supplementary Figure 7). Phylogenetic analysis was conducted with LlTYDC homologs from taxonomically diverse plant species using neighbor-joining phylogenetic reconstruction methods in order to infer the evolutionary history of LlTYDC. LlTYDC is a conserved homolog of the LaTYDC1 group (Supplementary Figure 7), exhibiting 90.41% amino acid sequence identity to those homologs (Wang et al., 2019).

### Subcellular Localization Analysis of LlTYDC

**FIGURE 8:**
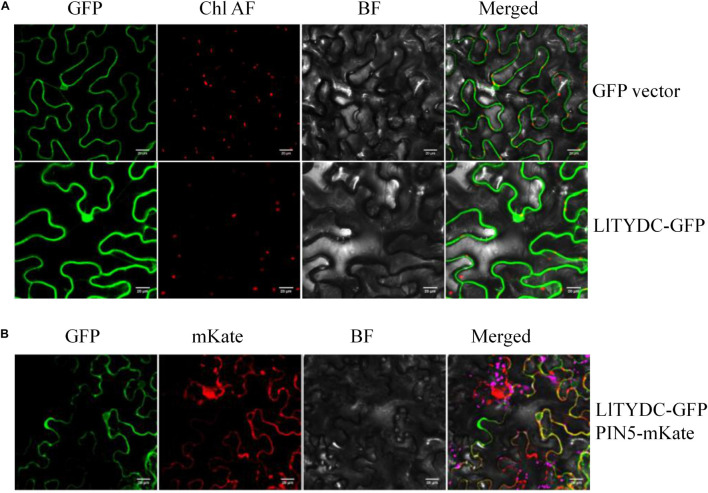
Subcellular localization of LlTYDC. **(A)** Subcellular localization of GFP alone and LlTYDC-GFP fusion protein in tobacco epidermal cells. **(B)** Tobacco epidermal cells co-expressing LlTYDC-GFP and an endoplasmic reticulum marker (PIN5-mKate). The photographs were taken in the green channel (GFP fluorescence), red channel (mKate fluorescence), combination of green and red channel, and bright field channel (BF) (*N* = 3, Scale bar = 20 μm).

To investigate the subcellular localization of LlTYDC, *Agrobacterium*-mediated infiltration of *N. benthamiana* leaves was conducted using LlTYDC fusions with enhanced GFP. In addition, the fluorescent marker protein that is characteristic for the endoplasmic reticulum (ER; mKate-PIN5) (Mravec et al., 2009), was co-expressed with LlTYDC-GFP. The LlTYDC-GFP fluorescence signals were present in the cytoplasm of mesophyll cells and also in the ER. Consequently, LlTYDC is likely localized in the cytoplasm and ER (Figure 8).

### LlTYDC Enzyme Activity Assays

**FIGURE 9:**
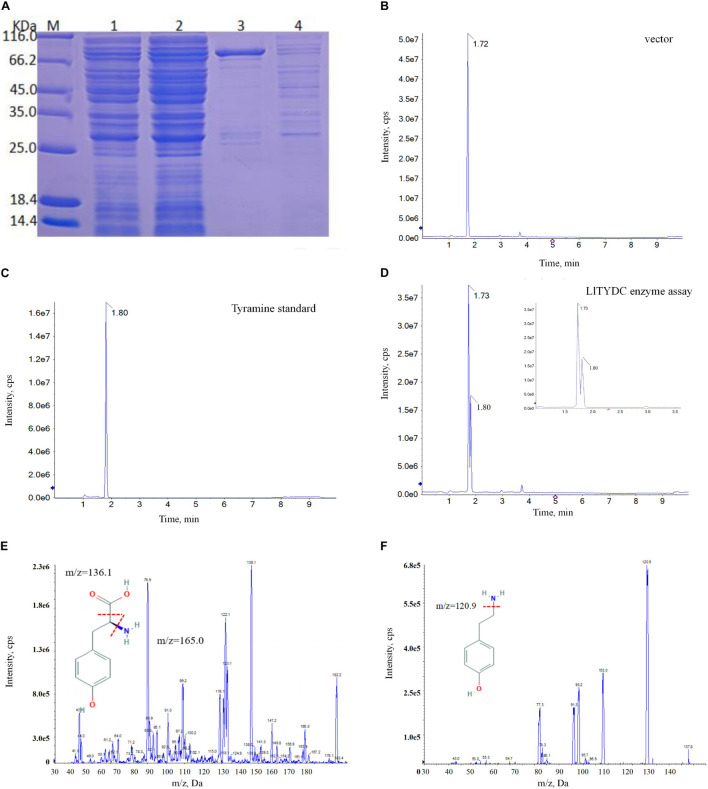
LlTYDC enzyme activity assays of recombinant LlTYDC. **(A)** SDS-PAGE gel image of LlTYDC expression in *E. coli*. The recombinant GST-tagged protein was purified from the culture lysates by affinity chromatography. 1: loading sample, 2: Flow through, 3: elution fractions, 4: wash through. M, molecular weight marker. **(B)** LC-MS/MS analysis of the enzyme assay of GST vector using tyrosine as a substrate. **(C)** LC-MS/MS analysis of the tyramine standard. **(D)** LC-MS/MS analysis of the enzyme assay of LlTYDC using tyrosine as a substrate. The reaction product tyramine was detected in an assay using recombinant LlTYDC. The identity was confirmed by MS/MS fragmentation of tyrosine **(E)** and tyramine **(F)**. The red line segment indicated the ion fragmentation pattern and the arrow indicated the retention time.

The first committed step at the beginning of isoquinoline alkaloid biosynthesis is the conversion of tyrosine into tyramine via catalysis with tyrosine decarboxylase (TYDC). The LaTYDC enzyme is a close homolog of LlTYDC and has been reported to play a key role in Gal biosynthesis (Wang et al., 2019). Consequently, the catalytic activity of LlTYDC was evaluated. The recombinant GST-tagged LlTYDC protein yielded a major band in SDS-PAGE, with the expected size of ∼83 kDa (Figure 9A). The purified protein was tested with tyrosine as a substrate and reaction assays indicated that LlTYDC exhibited catalytic activity yielding tyramine as the major reaction product (Figures 9B–F).

### Metabolomic Analysis of *L. longituba* Seedlings Treated With MeJA

To further clarify the effects of MJ treatment on metabolite accumulation, metabolites were extracted from *L. longituba* seedlings from the 75 μM MeJA (MJ-75) and 0 μM MeJA (MJ-0) treatments after 7 days of cultivation and by using six replicates. The metabolites were analyzed by GC-MS and LC-MS, resulting in clear differences between the MJ-75 and MJ-0 group plants. A total of 353 and 4,385 metabolites were identified from the GC-MS and LC-MS analyses, respectively (Supplementary Tables 11, 12). The metabolites identified from GC-MS analysis primarily included amino acids, lipids, nucleosides, and organic acids. In contrast, secondary metabolites like phenylpropanoids, quinones, flavonoids, terpenoids, steroids, and alkaloids were detected with LC-MS.

To visualize variation in metabolite profiles, PCA (R^2^X = 0.732 for GC-MS and R^2^X = 0.633 for LC-MS) and OPLS-DA (R^2^X = 0.671, R^2^Y = 0.999, *Q*^2^ = 0.987 for GC-MS and R^2^X = 0.742, R^2^Y = 0.999, *Q*^2^ = 0.978 for LC-MS) models were applied to the GC-MS and LC-MS profile data. The PCA score plot of the GC-MS (Supplementary Figure 8A) and LC-MS (Supplementary Figure 8C) profiles indicated that the first two principal components (PCs) explained 47.2 and 18.9% of the variance for the GC-MS analysis in addition to 40.0 and 14.0% of the variance for the LC-MS data, respectively. All MJ-0 samples clustered together in the ordinations and MJ-0 samples were significantly separated from the MJ-75 treatment samples, indicating that MJ treatment may strongly impact *L. longituba* seedling metabolomes. The OPLS-DA models yielded similar results to those of PCA (Supplementary Figures 8B,D). The R^2^X and Q^2^ metrics were used to evaluate the quality of the models (Supplementary Figure 9). The intercept (R^2^ and Q^2^ when the correlation coefficient is zero) was correlated with the extent of overfitting rather minimally (*R*^2^ = 0.968, *Q*^2^ = −0.215 for GC-MS and *R*^2^ = 0.894, *Q*^2^ = −0.433 for LC-MS) suggesting that the model was satisfactory.

**FIGURE 10:**
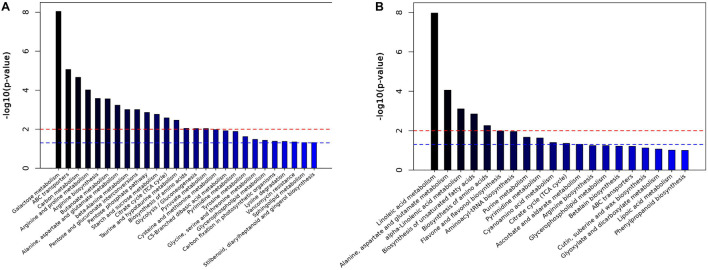
Histogram of pathway analysis in GC-MS **(A)** and LC-MS **(B)**. The horizontal blue line indicated *p* < 0.05; the horizontal red line indicated *p* < 0.01 (*N* = 6).

To better evaluate changes in metabolite abundances, a total of 103 and 669 differentially abundant metabolites were obtained from the GC-MS and LC-MS datasets based on the OPLS-DA model and metabolite VIP and *p* values (Supplementary Tables 13, 14). Clear dynamic changes were observed between the MJ-75 and MJ-0 samples based on heat map visualization (Supplementary Figure 10). To better understand the mechanism of metabolic pathway changes among different samples, pathway enrichment analysis of differential metabolites was performed. Galactose metabolism, ABC transporters, carbon metabolism, arginine and proline metabolism, and arginine biosynthesis were the five most enriched pathways based on the GC-MS analysis (Figure 10A). In addition, linoleic acid metabolism; alanine, aspartate, and glutamate metabolism; alpha-linoleic acid metabolism; biosynthesis of unsaturated fatty acids; and biosynthesis of amino acids were the five most enriched pathways based on LC-MS analysis (Figure 10B). Other pathways including starch and sucrose metabolism, flavone and flavonol biosynthesis, tyrosine metabolism, and phenylpropanoid biosynthesis were also affected by MJ treatment (Figure 10).

### Effects of MeJA on JA Synthesis in *L. longituba* Seedlings

**FIGURE 11:**
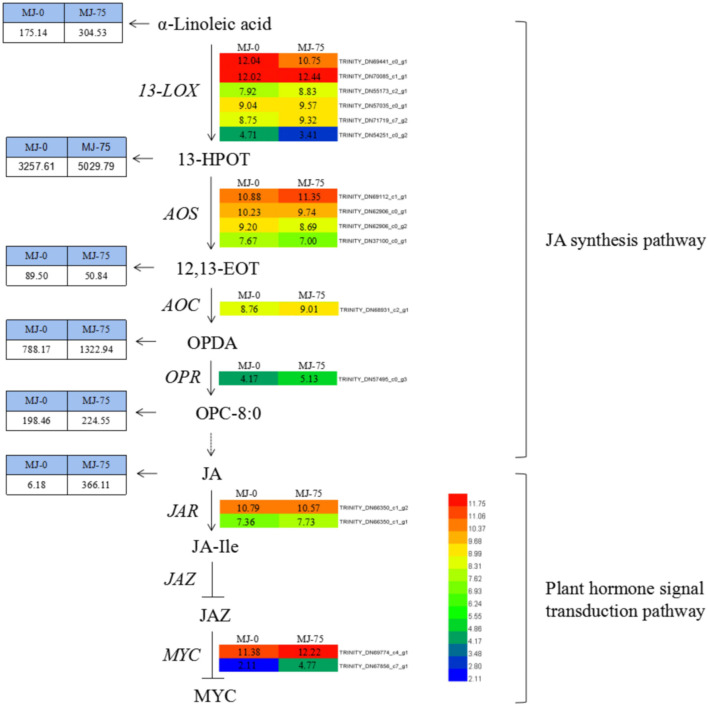
DEGs and metabolites involved in the JA synthesis and plant hormone signal transduction pathway. Heat maps showed the normalized gene expression values which represent the means ± SD of three biological replicates (*N* = 3). Metabolites contents showed the means ± SD of six biological replicates (*N* = 6). Gene expression values are presented as FPKM normalized log2-transformed counts. JA, jasmonic acid; MeJA, methyl jasmonate; 13-LOX, lipoxygenase; 13-HPOT, 13-hydroxy linolenic acid; AOS, allene oxide synthase; 12, 13-EOT, 12, 13-epoxyoctadecatrienoic acid; AOC, allene oxide cyclase; OPDA, 12-oxo-phytodienoic acid; OPR, 12-oxo-phytodienoic acid reductase; OPC-8:0, 3-oxo-2-(2(Z)-pentenyl)-cyclopentane-1 octanoic acid; JAR, jasmonic acid-amino acid synthase; JA-Ile, jasmonic acid-isoleucine; JAZ, jasmonic acid zimo-domain.

Methyl jasmonate treatment resulted in differential expression of 16 DEGs involved in JA biosynthesis and plant hormone signaling pathways. These included six types of 13-LOX (TRINITY_DN69441_c0_g1, TRINITY_DN70085_c1_g1, TRI NITY_DN55173_c2_g1, TRINITY_DN57035_c0_g1, TRINITY _DN71719_c7_g2, TRINITY_DN54251_c0_g2), four types of AOS (TRINITY_DN69112_c1_g1, TRINITY_DN62906_c0_g1, TRINITY_DN62906_c0_g2, TRINITY_DN37100_c0_g1), AOC (TRINITY_DN68931_c2_g1), and OPR (TRINITY_DN57495 _c0_g3) within the JA synthesis pathway. AOC and OPR genes were up-regulated. In addition, the LOX (four up-regulated and two down-regulated) and AOS (one up-regulated and three down-regulated) genes exhibited differential expression among groups (Figure 11). DEG transcripts related to JA signaling, including JAR (TRINITY_DN66350_c1_g2, TRINIT Y_DN66350_c1_g1) and the transcription factor MYC (TRIN ITY_DN69774_c4_g1, TRINITY_DN67856_c7_g1) exhibited increased expression in MJ-75 plants compared with MJ-0 plants (Figure 11). Interestingly, the metabolites in the JA synthesis pathway were also affected by MeJA treatment. α-Linoleic acid, 13-HPOT, OPDA, OPC-8:0, and JA exhibited 1. 74-, 1. 54-, 1. 68-, 1. 13-, and 59.24-fold upregulation, respectively, in MJ-75 plants compared with MJ-0 plants, while only 12, 13-EOT was downregulated in response to MeJA treatment.

### Effects of MeJA on Alkaloid Accumulations in *L. longituba* Seedlings

Among the 103 metabolites identified by GC-MS analysis, 42 were upregulated in MJ-75 samples, while 61 were down-regulated. Among the 669 metabolites identified by LC-MS analysis, 422 metabolites exhibited higher levels in MJ-75 plants, while 247 exhibited lower levels (Supplementary Tables 13, 14). Of these differentially abundant metabolites, 26 alkaloids and their derivatives were identified. Among these, upregulation of canthiumine (105,426.55-fold), sinomenine (7.54-fold), and erythratine (2.54-fold) was observed in the MJ-75 plants. In contrast, sanguinine, codeine-6-glucuronide, mescaline, and rosmarinine exhibited lower levels in the MJ-75 samples and the levels of these metabolites were 0.18–0.76-fold lower than in MJ-0 samples, respectively. More importantly, Amaryllidaceae alkaloids (including derivatives and intermediates) like amaryllisine, galanthamine, 93.-(-)-8-demethylmaritidine, narciclasine, norgalanthamine, lycoramine, narcissidine, lycoricidine, crinamine, lycorine, 11-hydroxyvittatine, and 4′-O-methylnorbelladine all accumulated in MJ-75 treatment plants (Table 1).

**TABLE 1:** Differential alkaloids and derivatives identified from LC-MS in MJ-75 and MJ-0 treatment *L. longituba* seedlings (*N* = 6).

Metabolites	VIP	FC MJ-75/MJ-0	*p*-value
Canthiumine	2.58	105426.55	***5.30E-08
Sinomenine	4.82	7.54	***3.00E-07
Erythratine	1.93	2.54	***6.21E-11
Amaryllisine	20.28	2.30	***2.34E-11
Galanthamine	9.29	2.25	***1.17E-10
Inuline	1.86	2.16	***3.15E-5
(-)-8-Demethylmaritidine	2.76	2.07	***2.74E-07
Normorphine	5.20	2.04	***7.75E-08
Narciclasine	2.15	1.79	***3.55E-05
Norgalanthamine	10.22	1.78	***2.64E-09
Lycoramine	14.85	1.56	***2.79E-08
Narcissidine	11.61	1.54	***2.68E-07
Lycoricidine	7.93	1.48	***8.09E-07
Crinamine	11.42	1.48	***1.31E-07
Senecionine	1.48	1.45	***5.62E-05
Methscopolamine	2.25	1.45	***3.66E-05
Dihydromorphine	1.42	1.43	***9.25E-08
Lycorine	9.06	1.41	**1.28E-03
11-Hydroxyvittatine	2.35	1.29	***3.99E-05
4′-O-Methylnorbelladine	2.08	1.15	*0.02
Hamayne	4.68	1.14	***4.21E-04
Trigonelline	1.12	1.08	*0.04
Sanguinine	6.04	0.76	***3.96E-05
Codeine-6-glucuronide	2.67	0.74	***9.58E-06
Mescaline	1.54	0.29	***4.41E-05
Rosmarinine	1.28	0.18	***1.17E-10

*P < 0.05, **P < 0.01, ***P < 0.001 by Student’s t-test.

**FIGURE 12:**
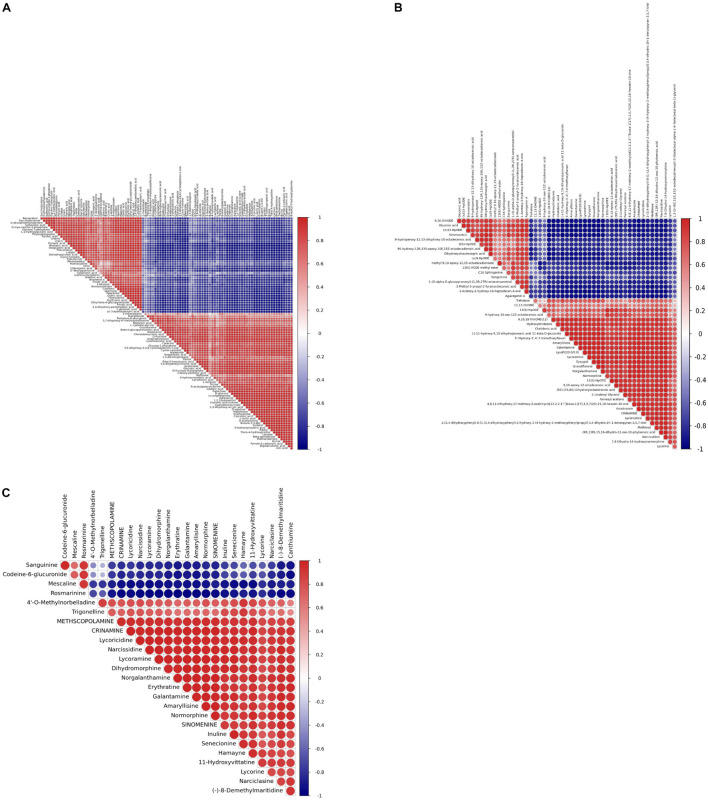
Heat map representing the r coefficients for the identified metabolites in *L. longituba* seedlings after MJ-75 and MJ-0 treatments. Correlations showed the 103 metabolites identified by GC-MS **(A)**, the top 50 statistical significance of all identified metabolites by LC-MS **(B)**, and statistical significance of alkaloids identified by LC-MS **(C)**. They are analyzed by using a Pearson correlation coefficient method. In the colored area, rectangles represent Pearson correlation coefficient (*r*) values of metabolites pair (*N* = 6).

Pairwise correlation analysis was performed using Pearson correlation coefficients to investigate the relationships between identified metabolites. A total of 103 correlations were calculated for the GC-MS profiles and the 50 highest correlations were calculated for the LC-MS profiles (Figures 12A,B and Supplementary Tables 15, 16). *r* values for GC-MS data correlations ranged from −0.989 for 2-picolinic acid and fumaric acid to 0.999 for triethanolamine and bicine, while *r* values ranged from −0.985 for (-)-11-hydroxy-9, 10-dihydrojasmonic acid 11-beta-D-glucoside and gluconic acid to 0.999 for 9, 10-epoxy-12-octadecenoic acid and (9Z)-(7S, 8S)-dihydroxyoctadecenoic acid based on LC-MS data.

Most of the Amaryllidaceae alkaloids exhibited high positive associations with each other, indicating that these metabolites may share similar biosynthetic pathways or exhibit similar changes in response to MJ-75 treatment (Figure 12C). In contrast, Amaryllidaceae alkaloids negatively correlated with the abundances of some carbohydrates and lipids. Galanthamine abundances were negatively correlated with those of gluconic acid (*r* = −0.982), but positively correlated with those of lycorine (*r* = 0.829), lycoricidine (*r* = 0.981), narcissidine (*r* = 0.986), crinamine (*r* = 0.986), norgalanthamine (*r* = 0.993), lycoramine (*r* = 0.995), and amaryllisine (*r* = 0.998) (Figure 12C and Supplementary Tables 16, 17).

**FIGURE 13:**
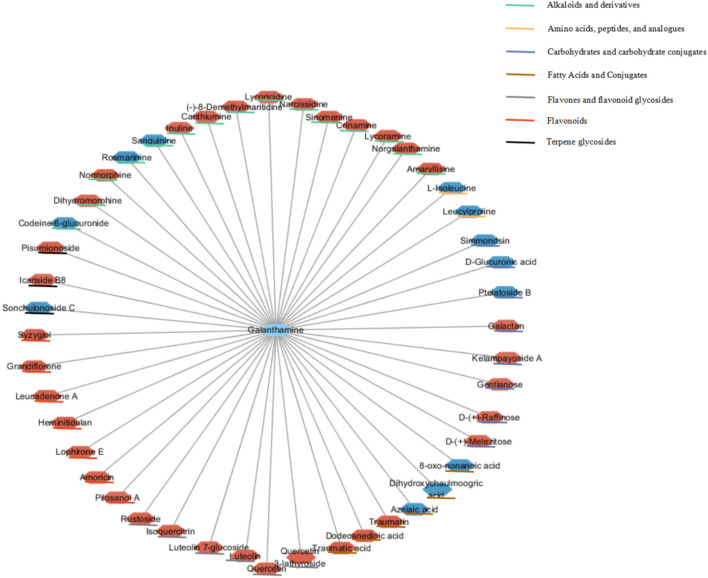
Correlation network based on identified galanthamine and other metabolites with significant correlations. Node colors represent different types of metabolites. Edges between nodes represent correlation identified as significant at | *r*| > 0.9, *p* < 0.001, where red and blue nodes represent positive and negative correlations (*N* = 6).

More importantly, 47 significant correlations were identified including 12 negative correlations and 35 positive correlations between galanthamine and other metabolites, with | *r*| values > 0.90 and *p* < 0.001 (Figure 13). Galanthamine abundances exhibited significant negative correlations with those of some amino acids, carbohydrates, fatty acids, but they were significantly and positively correlated with the abundances of most of the flavones, flavonoids, and alkaloids. The data presented here provides correlative information that may allow the identification of associations between the biosynthesis pathways of carbohydrates, amino acids, fatty acids, flavonoids, and alkaloids.

## Discussion

Methyl jasmonate can effectively induce the accumulation of Gal in *Lycoris* spp. like *L. chinensis* and *Lycoris aurea* (Mu et al., 2009; Wang et al., 2017). However, our understanding of MeJA-mediated regulation of Gal biosynthesis is limited in *L. longituba*. Consequently, clarification of the MeJA-mediated regulation mechanism of Gal biosynthesis is needed.

*Lycoris longituba* seedlings exhibited maximum Gal, Lyc, and Lycm levels after 75 μmol of MeJA treatment for 7 days. The accumulation of Gal, Lyc, and Lycm in *L. chinensis* was induced by 100, 50, and 50 μmol MeJA treatments after the 10th, 10th, and 3rd day of cultivation, respectively (Mu et al., 2009). Further, the accumulation of Gal in *L. aurea* increased due to treatment with 100 μmol MeJA for 6 h (Wang et al., 2017). The above results indicate that MeJA effects on alkaloid accumulation in *Lycoris* spp. differ among different species and plant ages. Higher activities of enzymes involved in nitrogen metabolism were observed after MeJA treatment, which may provide more materials for nitrogen-containing alkaloids. Similar effects of MeJA on nitrogen metabolism were reported for *Atropa belladonna* (Liu et al., 2019).

To further investigate the molecular mechanisms underlying MeJA-mediated Gal biosynthesis, MeJA-treated *L. longituba* seedlings (MJ-75, 7th day) and mock-treated seedlings (MJ-0, 7th day) were subjected to RNA-seq analysis to evaluate their transcriptional responses to MeJA treatment. A total of 185,442 unigenes were identified in *L. longituba*, while 141,111 unigenes were previously generated for *L. aurea*. Due to the large size of *Lycoris* genomes and the lack of available genome sequences for *Lycoris* spp., the sequence reads were not aligned to a reference genome for further analysis (Jiang et al., 2017; Li Q. et al., 2020). Among these unigenes, 4,548 were differentially expressed in response to MeJA treatment. Further, DEGs involved in the JA biosynthesis, JA signaling, and Gal biosynthesis pathways were identified, suggesting that both the Gal and JA pathways were affected by MeJA treatment in *L. longituba* seedlings. Previous transcriptome study in *L. aurea* investigated the MeJA-responsive transcriptional changes, and identified 4,165 DEGs belonging to 121 KEGG pathways between MeJA treated and non-treated samples. DEGs involved in 12 secondary metabolism pathways under MeJA were studied, and indicated that an extensive transcriptional reprogramming of secondary metabolites biosynthesis was triggered by MeJA treatment (Wang et al., 2017). Previous studies demonstrated that the application of MeJA induced the accumulation of different secondary metabolites in several plant species, including *Lycoris chinensis* (Mu et al., 2009), *Dendrobium officinale* (Jiao et al., 2018), *Physalis angulata* L. (Zhan et al., 2020), *Isatis tinctoria* L. (Gai et al., 2019), and *Vitis labrusca* L. (Moro et al., 2020). One major regulatory mechanism of MeJA-mediated secondary metabolite production in many plants is through the control of pathway related genes (Liu et al., 2018; Li et al., 2019a; Li W. et al., 2020). For example, the expression of some genes in terpenoid indole alkaloid (TIAs) pathways was induced by MeJA treatment in *Catharanthus roseus* (Pan et al., 2018). In this study, numerous predicted genes involved in a variety of metabolic pathways and genes encoding transcription factors were induced by MeJA application (Figures 5–7, 10). Interestingly, genes and metabolites known to be important components of the Gal synthesis pathway (Singh and Desgagné-Penix, 2014; Li Q. et al., 2020), including *NBS*, *OMT*, 4-hydroxycinnamic acid, 3,4-dihydroxycinnamic acid, 3,4-dihydroxybenzaldehyde, norbelladine, 4′-O-methylnorbelladine, N-demethylgalanthamine, and galanthamine, were all induced by MJ-75 treatment (Figure 6). Similar results could be observed in *L. aurea*, the expression of homologous gene of NpN4OMT in *L. aurea* showed more than two times higher under MeJA treatment (Wang et al., 2017). Amaryllidaceae alkaloid (including Gal) biosynthetic pathways can be divided into five stages (Desgagné-Penix, 2020). In this study, most gene expression in the phenylpropanoid pathway stage were unchanged or downregulated by MeJA treatment. However, genes in the core pathway stage were up-regulated by MeJA treatment except *TYDC*. TYDC catalyzes the conversion from tyrosine to tyramine in the core Gal biosynthesis pathway. *LaTYDC1* has been characterized in *L. aurea* and was found to be induced by MeJA (Wang et al., 2019). In this study, a *LlTYDC* gene was isolated and functionally characterized, revealing it to be a close homolog of LaTYDC1 with similar functionality. We speculate that the differential responses of *TYDC* to MeJA may be attributable to species-specific differences, plant ages, treatment periods, or other feedback inhibition mechanisms. The above results indicated that one regulatory mechanism of MeJA-mediated Gal production might be through the control of Gal biosynthetic pathway related genes.

Methyl jasmonate treatment not only induces the expression of genes involved in JA biosynthetic pathways, but it also can induce JA signaling pathways (Liu et al., 2018; Li W. et al., 2020). Similar results were observed in the present study, wherein RNA-seq and metabolite profiling suggested that the expression of putative JA biosynthesis genes (*AOC* and *OPR*) and JA signaling pathway genes (*JAR* and *MYC*) were upregulated. Moreover, metabolites including α-Linoleic acid, 13-HPOT, OPDA, OPC-8:0, and JA were induced, suggesting that exogenous application of MeJA could mediate JA biosynthesis and JA signaling pathway activity, that may in turn regulate a series of downstream genes in *L. longituba* seedlings. Transcription factors (TFs), including the MYB, MYB-related, WRKY, and bHLH families, have been reported to play key regulatory roles in plant growth, development, and secondary metabolite biosynthesis. TFs regulated the transcription of genes in different pathways to improve the production of secondary metabolites (Park et al., 2008; Hichri et al., 2010; Xie et al., 2016; Yin et al., 2017; Yu et al., 2018). In this study, 29,056 unigenes encoded putative TFs belonging to 58 TF families were found. A lot of TFs including bHLH, ERF, MYB-related families were up-regulated in response to MeJA treatment, suggesting their possible involvement in the regulation of secondary metabolites biosynthesis in *L. longituba.* In the future, further analysis of their expression changes may reveal their key functions in Gal accumulation.

Taken together, the above results indicate that MeJA can control the expression of some key genes in the Gal pathway that may in turn regulate the accumulation of pathway-related metabolites, and ultimately lead to the accumulation of Gal. The abundances of most flavones, flavonoids, flavonoid glycosides, alkaloids, and their derivatives exhibited high positive associations with Gal, and exhibited similar changes in response to MeJA treatment. Thus, the data presented here provide correlative information that may allow the elucidation of associations between different biosynthesis pathways. Future studies identifying the functional genes or transcription factors involved in Gal biosynthesis within *L. longituba* in response to MeJA will help us better understand MeJA-mediated regulatory mechanisms.

## Conclusion

In this study, transcriptome sequencing, GC-MS, and LC-MS analyses were used to investigate the effects of exogenous MeJA application on galanthamine (Gal) synthesis in *L. longituba*. A large dataset comprising unigenes and metabolites was generated, providing a rich resource to study secondary metabolite pathways. Genes and metabolites involved in Gal biosynthesis, JA biosynthesis, and JA signaling pathways were specifically evaluated. In addition, the *LlTYDC* gene was cloned from *L. longituba* and functionally characterized for the first time. These candidate genes and metabolites that responded to MeJA treatment could provide useful targets to study and better elucidate the regulatory mechanisms of Gal biosynthesis after MeJA treatment of *L. longituba* seedlings.

## Data Availability Statement

The original contributions presented in the study are publicly available. This data can be found here: National Center for Biotechnology Information (NCBI) BioProject database under accession number PRJNA720237.

## Author Contributions

QL, YZa, and YC designed the research. QL got the fundings and wrote the manuscript. QL, JX, and YZe performed the research. All authors contributed to the article and approved the submitted version.

## Conflict of Interest

QL was employed by the company Shanghai Co-Elite Agricultural Sci-Tech (Group) Co., Ltd. The remaining authors declare that the research was conducted in the absence of any commercial or financial relationships that could be construed as a potential conflict of interest.

## Publisher’s Note

All claims expressed in this article are solely those of the authors and do not necessarily represent those of their affiliated organizations, or those of the publisher, the editors and the reviewers. Any product that may be evaluated in this article, or claim that may be made by its manufacturer, is not guaranteed or endorsed by the publisher.

## Footnotes

1
http://www.geneontology.org

2
http://www.kegg.jp/

3
http://eggnog.embl.de

4
http://www.clustal.org/omega/

5
https://www.megasoftware.net/

6
http://www.metaboanalyst.ca/MetaboAnalyst/

7
http://www.hmdb.ca/

8
http://www.lipidmaps.org/
