# Supplementary material for: Transcriptomic and Metabolomic Analyses Reveals That Exogenous Methyl Jasmonate Regulates Galanthamine Biosynthesis in Lycoris longituba Seedlings
Source: Front Plant Sci. 2021 Sep 30;12:713795. doi: 10.3389/fpls.2021.713795 (PMC8514708; doi:10.3389/fpls.2021.713795)
Supplement: Supplementary file 1 [file Data_Sheet_1.docx]

Supplementary Material

# Supplementary Tables

**Supplementary Table 1** | Primers used in this study.

| **Primer name** | **Sequences** | **Application** |
| --- | --- | --- |
| PAL--F | CAAAGTGCAGAGCAACATAATCAAG | qRT-PCR analysis |
| PAL-R | TTCACTGTGCTCTTCAAATTCTCC | qRT-PCR analysis |
| C4H-F | GTCAGAGGAATCTCGTAGTCGTGTC | qRT-PCR analysis |
| C4H-R | CTCACCGTACACTGTAAAGACCATG | qRT-PCR analysis |
| C3H-F | CAGGTGCTTCGCCGAGTGG | qRT-PCR analysis |
| C3H-R | CCTCACCTTCACGTAGTGGG | qRT-PCR analysis |
| TYDC-F | CTGCGAGAGACAAAGTGTTGAATA | qRT-PCR analysis |
| TYDC-R | TACCTTTCGTAGCTCTTCAGGATT | qRT-PCR analysis |
| NBS-F | CCCAGGCATTCCAGGTATAA | qRT-PCR analysis |
| NBS-R | TACGACGATGGTGTCCTTCA | qRT-PCR analysis |
| OMT-F | AAGCTTGTCAGGGTTGGAGG | qRT-PCR analysis |
| OMT-R | TACACTCCTCCTCTTCCGGA | qRT-PCR analysis |
| CYP96T1-F | TGCTATGGCGAGGATGAAGG | qRT-PCR analysis |
| CYP96T1-R | ACATGTCCCTTCACCATCTG | qRT-PCR analysis |
| Actin-F | CATCCCTCAGCACCTTCCAG | qRT-PCR analysis |
| Actin-R | CTGGGATGCAAAAACCGCC | qRT-PCR analysis |
| RACE- TYDC-F1 | TTCGCGATGGTCTGTTTCCGGTT | 3’ RACE |
| RACE- TYDC-F2 | AGACCAAACTGGGCCGGAGTTAGACTG | 3’ RACE |
| *LlTYDC-F3* | atgggcagtcttggccctga | LlTYDC CDS |
| *LlTYDC-R3* | ttaattccttaatttataat | LlTYDC CDS |
| LlTYDC-GFP-F | cgatGGTCTCacaacatgggcagtcttggccctgataatattgctgagcttgaagcaaa | LlTYDC-GFP vector |
| LlTYDC-GFP-R | cagtGGTCTCatacaattccttaatttataatcttctccatgtgtgttcatcaaagcct | LlTYDC-GFP vector |
| LlTYDC-Protein-F | GACACGGATCCATGGGCAGTCTTGGCCCTGATAATATT | LlTYDC protein expression |
| LlTYDC-Protein-R | GTGTCCTCGAGTTAATTCCTTAATTTATAATCTT | LlTYDC protein expression |

C3H, p-coumarate 3-hydroxylase; C4H, cinnamate 4-hydroxylase; CYP96T1, noroxomaritidine synthase; GFP, fluorescent protein; NBS, norbelladine synthase; NMT, N-methyltransferase; OMT, norbelladine 4'-O-methyltransferase; PAL, phenylalanine ammonia-lyase; RT-qPCR, real-time quantitative polymerase chain reaction; TYDC, tyrosine decarboxylase.

**Supplementary Table 2** | Summary of the Illumina sequencing and reads assembly for *L. longituba* (*N*=3).

|  | **Contig** | **Transcript** | **Unigene** |
| --- | --- | --- | --- |
| Total length (bp) | 166556093 | 315474623 | 113874384 |
| Sequence No. | 610548 | 416829 | 185442 |
| Max. length (bp) | 14725 | 13465 | 13465 |
| Mean length (bp) | 272 | 756 | 614 |
| N50 (bp) | 337 | 1186 | 873 |
| N50 sequence No. | 101093 | 77394 | 33549 |
| N90 (bp) | 127 | 308 | 262 |
| N90 sequence No. | 461853 | 289385 | 135802 |
| GC% | 39.06 | 40.22 | 39.35 |

**Supplementary Table 3** | Summary of the annotation for *L. longituba* (*N*=3).

| **Database** | **Number** | **Percentage** |
| --- | --- | --- |
| NR | 52775 | 28.46 |
| KEGG | 19923 | 10.74 |
| eggNOG | 50289 | 27.12 |
| Swissprot | 35831 | 19.32 |
| In all database | 4337 | 2.34 |
| In at least one database | 54932 | 29.62 |

**Supplementary Table 4** | TF family.

| **Family** | **Count** |
| --- | --- |
| bHLH | 2406 |
| ERF | 2348 |
| MYB_related | 1909 |
| NAC | 1885 |
| TCP | 1512 |
| M-type_MADS | 1339 |
| C2H2 | 1336 |
| MYB | 1214 |
| WRKY | 1130 |
| B3 | 1059 |
| FAR1 | 967 |
| G2-like | 922 |
| LBD | 899 |
| Trihelix | 789 |
| HD-ZIP | 757 |
| bZIP | 748 |
| C3H | 747 |
| GRAS | 602 |
| CAMTA | 420 |
| NF-YB | 414 |
| NF-YA | 366 |
| ARF | 365 |
| NF-YC | 334 |
| SBP | 323 |
| GATA | 318 |
| HB-other | 318 |
| HSF | 306 |
| E2F/DP | 289 |
| MIKC_MADS | 243 |
| Nin-like | 222 |
| ZF-HD | 199 |
| BES1 | 168 |
| Dof | 161 |
| CO-like | 156 |
| TALE | 155 |
| RAV | 143 |
| WOX | 143 |
| GeBP | 133 |
| STAT | 130 |
| DBB | 129 |
| ARR-B | 126 |
| AP2 | 110 |
| CPP | 106 |
| S1Fa-like | 104 |
| YABBY | 99 |
| BBR-BPC | 84 |
| GRF | 62 |
| NF-X1 | 57 |
| HB-PHD | 51 |
| SRS | 47 |
| Whirly | 44 |
| LSD | 43 |
| EIL | 33 |
| HRT-like | 30 |
| VOZ | 26 |
| SAP | 21 |
| LFY | 6 |
| NZZ/SPL | 3 |

**Supplementary Table 5** | Overview of GO annotation.

In the attachment.

**Supplementary Table 6** | Overview of eggNOR annotation.

| **Description** | **number** | **category** |
| --- | --- | --- |
| A:RNA processing and modification | 1939 | A |
| B:Chromatin structure and dynamics | 1112 | B |
| C:Energy production and conversion | 1406 | C |
| D:Cell cycle control, cell division, chromosome partitioning | 842 | D |
| E:Amino acid transport and metabolism | 1333 | E |
| F:Nucleotide transport and metabolism | 412 | F |
| G:Carbohydrate transport and metabolism | 2146 | G |
| H:Coenzyme transport and metabolism | 574 | H |
| I:Lipid transport and metabolism | 1052 | I |
| J:Translation, ribosomal structure and biogenesis | 1765 | J |
| K:Transcription | 2792 | K |
| L:Replication, recombination and repair | 4630 | L |
| M:Cell wall/membrane/envelope biogenesis | 555 | M |
| N:Cell motility | 2 | N |
| O:Posttranslational modification, protein turnover, chaperones | 3466 | O |
| P:Inorganic ion transport and metabolism | 1184 | P |
| Q:Secondary metabolites biosynthesis, transport and catabolism | 1339 | Q |
| R:General function prediction only | 18594 | R |
| S:Function unknown | 12090 | S |
| T:Signal transduction mechanisms | 4122 | T |
| U:Intracellular trafficking, secretion, and vesicular transport | 1485 | U |
| V:Defense mechanisms | 283 | V |
| W:Extracellular structures | 91 | W |
| Y:Nuclear structure | 136 | Y |
| Z:Cytoskeleton | 663 | Z |

**Supplementary Table 7** | KEGG pathway analysis.

In the attachment.

**Supplementary Table 8** | Metabolic pathways and their related number of DEGs in the MJ-75 samples as compared with MJ-0 (*N*=3).

| **PathwayID** | **Pathway** | **Up number** | **Down number** | **DEG number** | **Total number** |
| --- | --- | --- | --- | --- | --- |
| ko00270 | Cysteine and methionine metabolism | 5 | 21 | 26 | 252 |
| ko00940 | Phenylpropanoid biosynthesis | 1 | 22 | 23 | 244 |
| ko00190 | Oxidative phosphorylation | 12 | 8 | 20 | 395 |
| ko00500 | Starch and sucrose metabolism | 8 | 12 | 20 | 334 |
| ko00010 | Glycolysis / Gluconeogenesis | 11 | 8 | 19 | 457 |
| ko00040 | Pentose and glucuronate interconversions | 0 | 19 | 19 | 157 |
| ko00520 | Amino sugar and nucleotide sugar metabolism | 3 | 16 | 19 | 280 |
| ko00230 | Purine metabolism | 2 | 16 | 18 | 259 |
| ko00195 | Photosynthesis | 3 | 12 | 15 | 136 |
| ko00240 | Pyrimidine metabolism | 1 | 13 | 14 | 158 |
| ko00030 | Pentose phosphate pathway | 5 | 7 | 12 | 181 |
| ko00480 | Glutathione metabolism | 4 | 8 | 12 | 193 |
| ko00564 | Glycerophospholipid metabolism | 5 | 7 | 12 | 281 |
| ko00620 | Pyruvate metabolism | 2 | 10 | 12 | 286 |
| ko00710 | Carbon fixation in photosynthetic organisms | 4 | 8 | 12 | 231 |
| ko00196 | Photosynthesis-antenna proteins | 0 | 11 | 11 | 48 |
| ko00860 | Porphyrin and chlorophyll metabolism | 5 | 6 | 11 | 86 |
| ko00260 | Glycine, serine and threonine metabolism | 4 | 6 | 10 | 174 |
| ko00051 | Fructose and mannose metabolism | 5 | 4 | 9 | 194 |
| ko00020 | Citrate cycle (TCA cycle) | 2 | 6 | 8 | 223 |
| ko00052 | Galactose metabolism | 2 | 6 | 8 | 177 |
| ko00061 | Fatty acid biosynthesis | 0 | 8 | 8 | 107 |
| ko00330 | Arginine and proline metabolism | 5 | 2 | 7 | 130 |
| ko00350 | Tyrosine metabolism | 2 | 5 | 7 | 99 |
| ko00561 | Glycerolipid metabolism | 6 | 1 | 7 | 139 |
| ko00400 | Phenylalanine, tyrosine and tryptophan biosynthesis | 2 | 4 | 6 | 133 |
| ko00630 | Glyoxylate and dicarboxylate metabolism | 2 | 4 | 6 | 193 |
| ko00950 | Isoquinoline alkaloid biosynthesis | 2 | 4 | 6 | 53 |
| ko00053 | Ascorbate and aldarate metabolism | 2 | 3 | 5 | 99 |
| ko00360 | Phenylalanine metabolism | 1 | 4 | 5 | 104 |
| ko00410 | beta-Alanine metabolism | 3 | 2 | 5 | 103 |
| ko00511 | Other glycan degradation | 2 | 3 | 5 | 160 |
| ko00740 | Riboflavin metabolism | 5 | 0 | 5 | 33 |
| ko00750 | Vitamin B6 metabolism | 4 | 1 | 5 | 21 |
| ko00780 | Biotin metabolism | 0 | 5 | 5 | 46 |
| ko00910 | Nitrogen metabolism | 2 | 3 | 5 | 69 |
| ko00941 | Flavonoid biosynthesis | 2 | 3 | 5 | 81 |
| ko00960 | Tropane, piperidine and pyridine alkaloid biosynthesis | 1 | 4 | 5 | 89 |
| ko00062 | Fatty acid elongation | 0 | 4 | 4 | 66 |
| ko00220 | Arginine biosynthesis | 3 | 1 | 4 | 109 |
| ko00250 | Alanine, aspartate and glutamate metabolism | 2 | 2 | 4 | 155 |
| ko00280 | Valine, leucine and isoleucine degradation | 2 | 2 | 4 | 130 |
| ko00300 | Lysine biosynthesis | 1 | 3 | 4 | 25 |
| ko00380 | Tryptophan metabolism | 3 | 1 | 4 | 122 |
| ko00450 | Selenocompound metabolism | 0 | 4 | 4 | 74 |
| ko00592 | alpha-Linolenic acid metabolism | 1 | 3 | 4 | 88 |
| ko00640 | Propanoate metabolism | 0 | 4 | 4 | 112 |
| ko00760 | Nicotinate and nicotinamide metabolism | 2 | 2 | 4 | 41 |
| ko00770 | Pantothenate and CoA biosynthesis | 2 | 2 | 4 | 58 |
| ko00920 | Sulfur metabolism | 0 | 4 | 4 | 67 |
| ko00071 | Fatty acid degradation | 2 | 1 | 3 | 122 |
| ko00261 | Monobactam biosynthesis | 1 | 2 | 3 | 23 |
| ko00290 | Valine, leucine and isoleucine biosynthesis | 0 | 3 | 3 | 48 |
| ko00310 | Lysine degradation | 2 | 1 | 3 | 120 |
| ko00460 | Cyanoamino acid metabolism | 1 | 2 | 3 | 83 |
| ko00531 | Glycosaminoglycan degradation | 0 | 3 | 3 | 39 |
| ko00603 | Glycosphingolipid biosynthesis - globo and isoglobo series | 0 | 3 | 3 | 14 |
| ko00650 | Butanoate metabolism | 0 | 3 | 3 | 64 |
| ko00100 | Steroid biosynthesis | 0 | 2 | 2 | 73 |
| ko00130 | Ubiquinone and other terpenoid-quinone biosynthesis | 0 | 2 | 2 | 106 |
| ko00340 | Histidine metabolism | 2 | 0 | 2 | 54 |
| ko00562 | Inositol phosphate metabolism | 0 | 2 | 2 | 127 |
| ko00565 | Ether lipid metabolism | 1 | 1 | 2 | 117 |
| ko00591 | Linoleic acid metabolism | 0 | 2 | 2 | 37 |
| ko00604 | Glycosphingolipid biosynthesis - ganglio series | 0 | 2 | 2 | 10 |
| ko00660 | C5-Branched dibasic acid metabolism | 0 | 2 | 2 | 19 |
| ko00670 | One carbon pool by folate | 0 | 2 | 2 | 51 |
| ko00900 | Terpenoid backbone biosynthesis | 0 | 2 | 2 | 128 |
| ko00904 | Diterpenoid biosynthesis | 1 | 1 | 2 | 36 |
| ko00945 | Stilbenoid, diarylheptanoid and gingerol biosynthesis | 0 | 2 | 2 | 31 |
| ko00073 | Cutin, suberine and wax biosynthesis | 1 | 0 | 1 | 113 |
| ko00510 | N-Glycan biosynthesis | 0 | 1 | 1 | 111 |
| ko00514 | Other types of O-glycan biosynthesis | 0 | 1 | 1 | 9 |
| ko00590 | Arachidonic acid metabolism | 0 | 1 | 1 | 29 |
| ko00600 | Sphingolipid metabolism | 0 | 1 | 1 | 63 |
| ko00601 | Glycosphingolipid biosynthesis - lacto and neolacto series | 0 | 1 | 1 | 2 |
| ko00730 | Thiamine metabolism | 0 | 1 | 1 | 54 |
| ko00785 | Lipoic acid metabolism | 0 | 1 | 1 | 14 |
| ko01040 | Biosynthesis of unsaturated fatty acids | 0 | 1 | 1 | 40 |

**Supplementary Table 9** | Differentially expressed transcription factors (TFs) in the MJ-75 samples as compared with MJ-0 (*N*=3).

| **TF Family** | **Count** | **Up** | **Down** |
| --- | --- | --- | --- |
| AP2 | 5 | 2 | 3 |
| ARF | 13 | 7 | 6 |
| ARR-B | 7 | 1 | 6 |
| B3 | 57 | 15 | 42 |
| BBR-BPC | 4 | 2 | 2 |
| BES1 | 9 | 0 | 9 |
| bHLH | 95 | 32 | 63 |
| bZIP | 32 | 11 | 21 |
| C2H2 | 63 | 21 | 42 |
| C3H | 34 | 9 | 25 |
| CAMTA | 11 | 10 | 1 |
| CO-like | 16 | 4 | 12 |
| CPP | 4 | 1 | 3 |
| DBB | 7 | 2 | 5 |
| Dof | 8 | 2 | 6 |
| E2F/DP | 16 | 11 | 5 |
| ERF | 126 | 59 | 67 |
| FAR1 | 31 | 8 | 23 |
| G2-like | 31 | 14 | 17 |
| GATA | 20 | 5 | 15 |
| GeBP | 10 | 3 | 7 |
| GRAS | 16 | 8 | 8 |
| GRF | 4 | 1 | 3 |
| HB-other | 8 | 4 | 4 |
| HB-PHD | 6 | 1 | 5 |
| HD-ZIP | 24 | 16 | 8 |
| HSF | 15 | 4 | 11 |
| LBD | 38 | 24 | 14 |
| MIKC_MADS | 8 | 1 | 7 |
| M-type_MADS | 36 | 21 | 15 |
| MYB | 44 | 19 | 25 |
| MYB_related | 67 | 22 | 45 |
| NAC | 119 | 28 | 91 |
| NF-YA | 16 | 7 | 9 |
| NF-YB | 21 | 7 | 14 |
| NF-YC | 17 | 9 | 8 |
| Nin-like | 9 | 2 | 7 |
| RAV | 8 | 4 | 4 |
| S1Fa-like | 9 | 0 | 9 |
| SBP | 7 | 3 | 4 |
| SRS | 4 | 2 | 2 |
| STAT | 10 | 5 | 5 |
| TALE | 5 | 4 | 1 |
| TCP | 59 | 54 | 5 |
| Trihelix | 35 | 11 | 24 |
| Whirly | 4 | 0 | 4 |
| WOX | 6 | 4 | 2 |
| WRKY | 48 | 12 | 36 |
| YABBY | 6 | 1 | 5 |
| ZF-HD | 3 | 3 | 0 |

**Supplementary Table 10** | Putative genes involved in Gal biosynthesis pathway.

| Name | Length (bp) | Annotation | Species | E-value | Similarity(%) | Accession No. |
| --- | --- | --- | --- | --- | --- | --- |
| PAL | 2127 | Phenylalanine ammonia-lyase | *Lycoris radiata* | 0 | 96.5 | TRINITY_DN68305_c0_g1 |
| TYDC | 1515 | Tyrosine decarboxylase | *Narcissus aff. pseudonarcissus* | 0 | 93.1 | TRINITY_DN69413_c0_g2 |
| C4H | 1509 | Cinammate 4-hydroxylase | *Lycoris radiata* | 0 | 98.5 | TRINITY_DN69319_c0_g5 |
| C3H | 1461 | p-coumarate 3-hydroxylase | *Narcissus pseudonarcissus* | 0 | 92.9 | TRINITY_DN64397_c5_g1 |
| NBS | 465 | norbelladine synthase | *Narcissus aff. pseudonarciss* | 7.80E-75 | 72.1 | TRINITY_DN61524_c0_g1 |
| OMT | 717 | Norbelladine 4'-O-methyltransferase | *Lycoris radiata* | 6.10E-176 | 95.4 | TRINITY_DN68071_c0_g2 |
| CYP96T1 | 1352 | Noroxomaritidine synthase | *Narcissus aff. pseudonarcissus* | 0 | 89.9 | TRINITY_DN59080_c0_g1 |

C3H, p-coumarate 3-hydroxylase; C4H, cinnamate 4-hydroxylase; CYP96T1, noroxomaritidine synthase; NBS, norbelladine synthase; NMT, N-methyltransferase; OMT, norbelladine 4'-O-methyltransferase; PAL, phenylalanine ammonia-lyase; TYDC, tyrosine decarboxylase.

**Supplementary Table 11** | GC-MS metabolic profiles analysis.

In the attachment.

**Supplementary Table 12** | LC-MS metabolic profiles analysis.

In the attachment.

**Supplementary Table 13** | Differential metabolites of GC-MS.

In the attachment.

**Supplementary Table 14** | Differential metabolites of LC-MS.

In the attachment.

**Supplementary Table 15** | Correlation analysis of GC-MS.

In the attachment.

**Supplementary Table 16** | Correlation analysis of LC-MS.

In the attachment.

**Supplementary Table 17** | Alkaloids correlation analysis.

In the attachment.

**Supplementary Table 18** | Annotation and expression of unigenes.

**Supplementary Table 19** | The assembled unigenes.

# Supplementary Figures

**Supplementary Figure 1**| Length distributions of the transcripts (A) and unigenes (B).

**Supplementary Figure 2**| Species homology analysis of the Nr annotation.


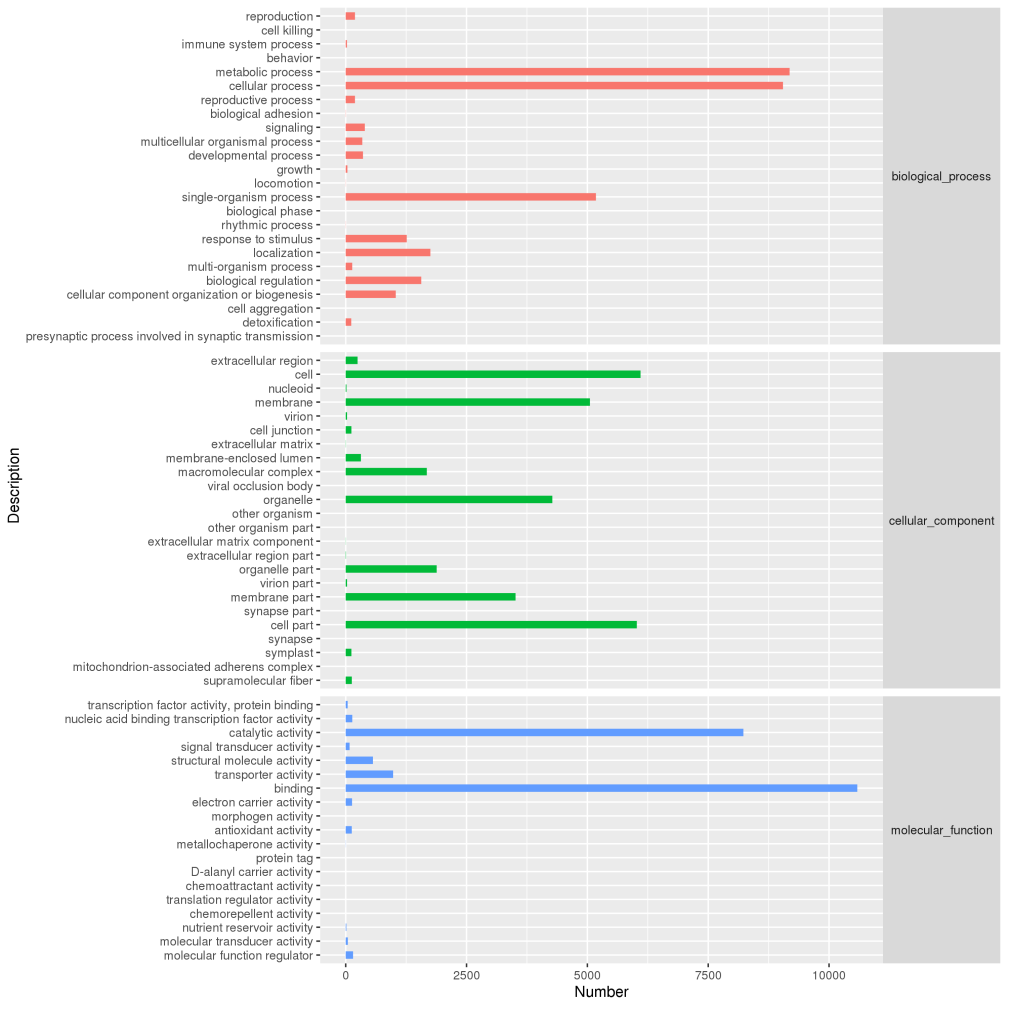


**Supplementary Figure 3**| Gene Ontology (GO) annotation.


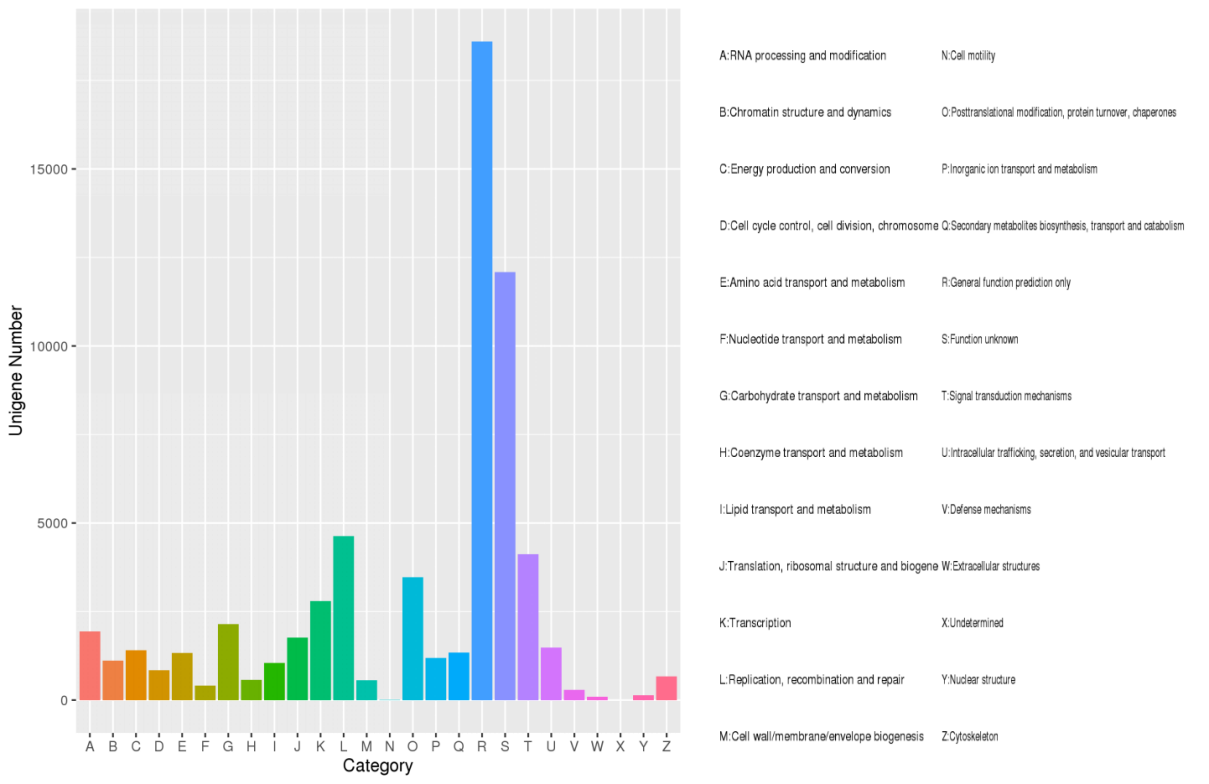


**Supplementary Figure 4**| eggNOG annotation.


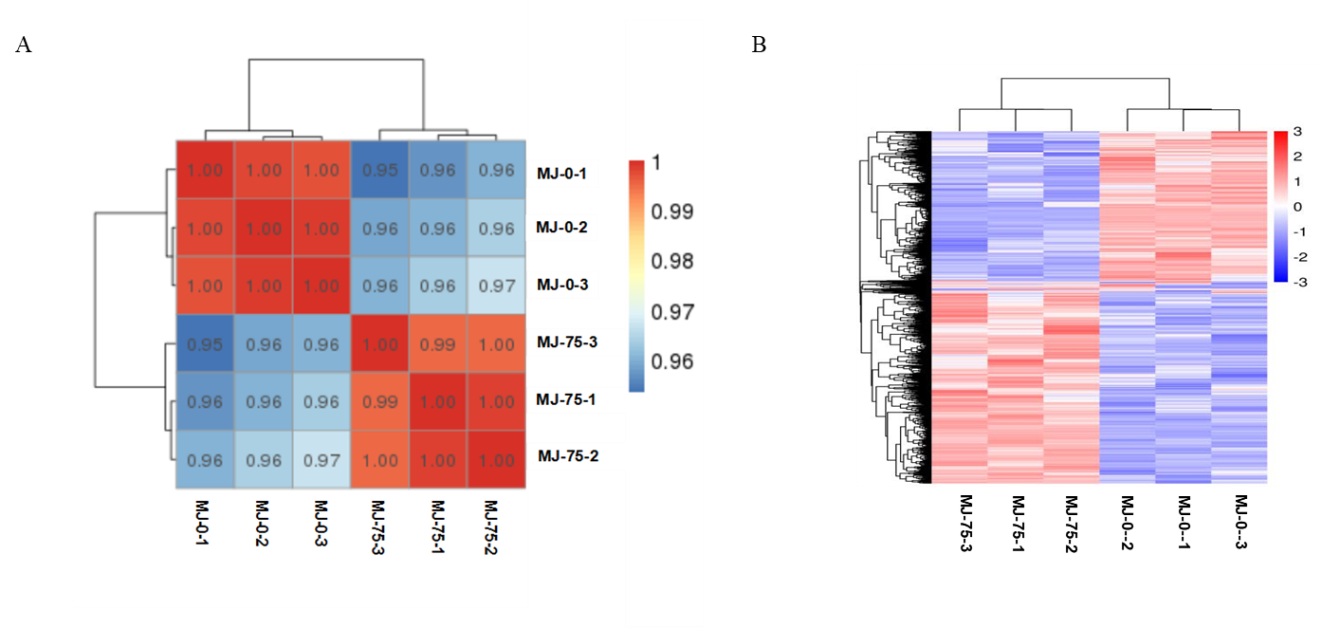
**Supplementary Figure 5**| Cluster (A) and heatmap (B) analysis of the differentially expressed genes (DEGs).


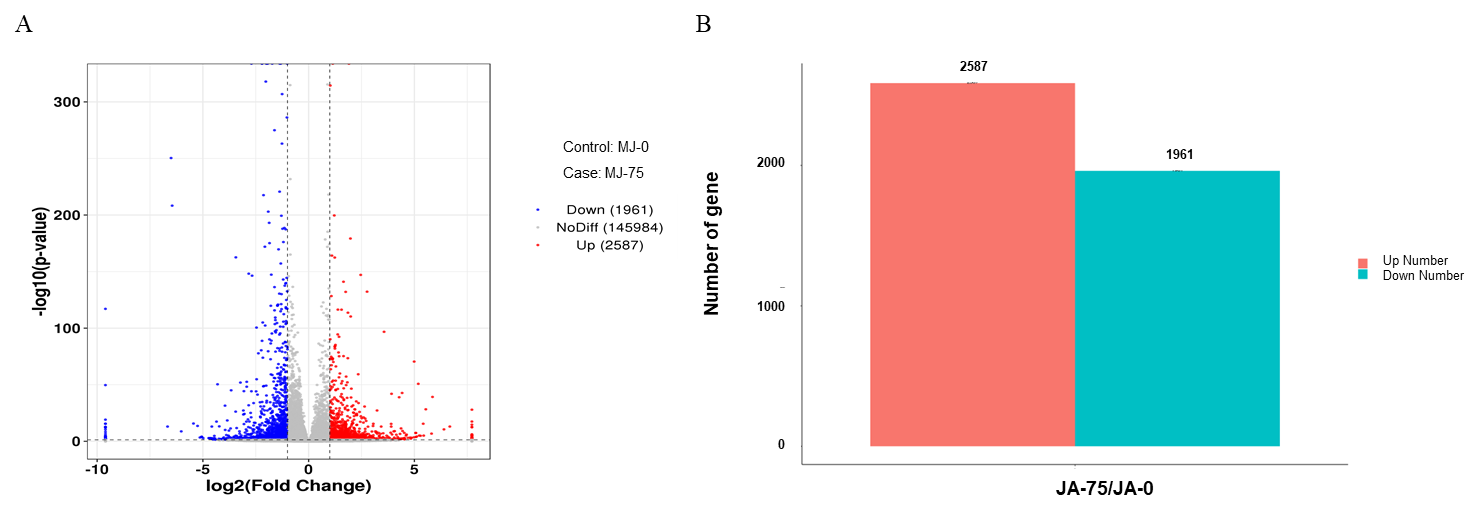


**Supplementary Figure 6**| Transcriptomes of *L. longituba* under MJ treatment. (A) The volcano map of differentially expressed genes (DEGs). Red spots represent up-regulated DEGs, green spots indicate down-regulated DEGs and gray spots are unigenes that did not change significantly under MJ-75 treatment. (B) Number of differentially expressed genes (DEGs) showing up- (red) or down- (green) regulation between the samples.

**Supplementary Figure 7**| Sequence analysis of LlTYDC. (a) Protein sequence alignment of LlTYDC with homologous proteins. (b) Phylogenetic analysis of LlTYDC and other TYDCs. Sequence alignment was performed with Clustal Omega, and the phylogenetic tree was constructed with MEGA version 5.2 using the neighbor-joining method with 1000 bootstrap replicates. Numbers at the nodes indicate the percentage bootstrap values.

**Supplementary Figure 8**| Principle component analysis (PCA) and orthogonal partial least squares-discriminant analysis (OPLS-DA) of metabolites derived from GC-MS and LC-MS data. (A, C) are the scores plots of PCA models, respectively. (B, D) are the S-plot of OPLS-DA models, respectively.

**Supplementary Figure 9**| Permutation test for the OPLS-DA model. The permutation plot displays the correlation coefficient between the original y-variable and the permuted y-variable on the X-axis versus the cumulative R2 and Q2 on the Y-axis, and the regression line was drawn for GC-MS (A) and LC-MS (B). The intercept is a measure of the overfit.

**Supplementary Figure 10**| Heatmap analysis of 103 and 669 identified differential metabolites between MJ-75 and MJ-0 *L. longituba* seedling by GC-MS(A) and LC-MS (B). The lowest ratios are in blue, and the highest ratio in red. The full lists of differential metabolites were shown in Supplementary Table 13 and Supplementary Table 14.
